# Resequencing of *cv* CRI‐12 family reveals haplotype block inheritance and recombination of agronomically important genes in artificial selection

**DOI:** 10.1111/pbi.13030

**Published:** 2018-12-03

**Authors:** Xuke Lu, Xiaoqiong Fu, Delong Wang, Junyi Wang, Xiugui Chen, Meirong Hao, Junjuan Wang, Kyle A. Gervers, Lixue Guo, Shuai Wang, Zujun Yin, Weili Fan, Chunwei Shi, Xiaoge Wang, Jun Peng, Chao Chen, Ruifeng Cui, Na Shu, Binglei Zhang, Mingge Han, Xiaojie Zhao, Min Mu, John Z. Yu, Wuwei Ye

**Affiliations:** ^1^ State Key Laboratory of Cotton Biology Key Laboratory for Cotton Genetic Improvement Institute of Cotton Research of Chinese Academy of Agricultural Sciences Ministry of Agriculture Anyang Henan China; ^2^ Hangzhou 1 Gene Technology CO., LTD Hangzhou Zhejiang China; ^3^ Crop Germplasm Research Unit Southern Plains Agricultural Research Center US Department of Agriculture—Agricultural Research Service (USDA‐ARS) College Station TX USA

**Keywords:** CRI‐12 family, SNPs, haplotype block, inheritance and recombination, artificial selection

## Abstract

Although efforts have been taken to exploit diversity for yield and quality improvements, limited progress on using beneficial alleles in domesticated and undomesticated cotton varieties is limited. Given the complexity and limited amount of genomic information since the completion of four cotton genomes, characterizing significant variations and haplotype block inheritance under artificial selection has been challenging. Here we sequenced *Gossypium hirsutum* L. *cv* CRI‐12 (the cotton variety with the largest acreage in China), its parental cultivars, and progeny cultivars, which were bred by the different institutes in China. In total, 3.3 million SNPs were identified and 118, 126 and 176 genes were remarkably correlated with *Verticillium wilt*, salinity and drought tolerance in CRI‐12, respectively. Transcriptome‐wide analyses of gene expression, and functional annotations, have provided support for the identification of genes tied to these tolerances. We totally discovered 58 116 haplotype blocks, among which 23 752 may be inherited and 1029 may be recombined under artificial selection. This survey of genetic diversity identified loci that may have been subject to artificial selection and documented the haplotype block inheritance and recombination, shedding light on the genetic mechanism of artificial selection and guiding breeding efforts for the genetic improvement of cotton.

## Introduction

*Gossypium* is a diverse genus consisting of at least 45 diploids and six allotetraploids with genome types A‐G and K found among its diploid species—which originated from the paleohexaploidization of a eudicot progenitor about 5–10 million years ago (MYA). At least six hybridized and chromosome‐doubled allotetraploid *Gossypium* species were descended from A and D diploids about 1.5 MYA (Li *et al*., 2014, 2015; Paterson *et al*., 2012). Cultivation of *Gossypium* began about 7000 years ago and the genus has subsequently been the target of heightened efforts to increase its fibre productivity, serving as an ideal model for studying fibre development and genome polyploidization (Jiang *et al*., 1998; Paterson *et al*., 2012; Yuan *et al*., 2015). The shrinking area for cotton planting and increasing demand for cotton cultivars with high‐yield and high‐quality fibre pose great challenges to cotton breeding.

CRI‐12—the cultivar most widely grown in China from 1989 to the present for its high yield, fibre quality and tolerance to different diseases in the Yangtze, the Huanghe and the Inland regions (the three major cotton‐production regions in China). It was developed from a cross between Xingtai6871 and Wuganda4 in 1984. CRI‐12 was bred in disease nursery and serious illness field, so high‐resistance to *Fusarium wilt* (index was 4.1) and *Verticillium wilt* (index was 14.3) were its main merits, and this exactly met the demand of multiresistance to different diseases in 80s in the 20th century. Investigation of disease‐resistance showed *F. wilt* index was always 2.0 in 2 years and the variation in *V. wilt* index was merely 3.2. Besides, statistic data indicated lint yield of CRI‐12 before frost stood the first or second place at 88/98 different places (89.8%). All these merits showed extensive adaptability and stability of CRI‐12.

CRI‐12 soon became the only cotton variety in 1990 to win the First Prize of National Invention Award. By now, CRI‐12–the most widely grown cotton variety in China with a planting area of 1.07 × 10^7^ ha and bred by the Institute of Cotton Research at the Chinese Academy of Agricultural Sciences–now encompasses more than half of all cotton production area in China (Tan and Liu, 2013). Because of the excellent performance of CRI‐12, it was frequently used for breeding new cotton varieties, including CRI35 (2.04 × 10^6^ ha), Yumian2067 (1.00 × 10^4^ ha), Lumianyan16 (5.53 × 10^5^ ha), Jinmian11 (1.85 × 10^5^ ha), Jinmian20 (3.33 × 10^3^ ha) and Jinmian33 (5.53 × 10^4^ ha), etc. Until now, progenies of CRI‐12 (using CRI‐12 as one of the parents) were most compared with other cotton varieties. Accordingly, it would be of great utility to evaluate the blocks inheritance of polymorphisms (spanning many agronomically important genes) during pedigree breeding for cotton breeding and improvement efforts. Documents showed linkage blocks (clusters of genes inherited together) were proposed before and verified in multiple recombinant individuals of rice (Jia *et al*., 2012; Wang *et al*., 2015), while Lai *et al*. (2010) also reported genome‐wide patterns of genetic variation in six elite maize inbred lines. Despite these advances, the role and mechanism of SNP haplotype inheritance in cotton breeding is still puzzling.

High‐quality assemblies of the tetraploid cotton (*Gossypium hirsutum* L.) genome (Li *et al*., 2015; Zhang *et al*., 2015) could provide accurate and effective sequence information for future resequencing efforts, with added utility of completed diploid (A and D) genomes (Li *et al*., 2014; Paterson *et al*., 2012; Wang *et al*., 2012). *Gossypium hirsutum* (AADD), the Upland cotton that accounts for more than 90% of world's commercial cotton production (Wendel, 1989), is the most widely grown cotton species among the four cultivated species. *Gossypium hirsutum* continues to serve as the most important natural fibre resource and as a significant oilseed. CRI‐12, one of the most successful Upland cotton varieties, was one of the cotton lines considered when developing the allotetraploid cotton genome sequence, especially for its high fibre yield, quality, disease tolerance (especially to *F. wilt* and *V. wilt*) and extensive adaptability and stability throughout China's cotton‐producing history. Much of the early work on cotton genomics focused on analyzing genome‐wide genetic variation to further investigate gene functions in cotton and to generate data and resources for cotton research–all while information on SNP block polymorphism inherited between parents and progeny was still unknown.

Sudden demographic expansions pose great challenges in cotton artificial selection, potentially affecting the entire genome (Luikart *et al*., 2003). Artificial selection could influence important loci variations affecting traits selected in the breeding process. The genomic functional architecture, that is, mutation and recombination rates are important factors in determining the genomic landscape of differentiation (Nachman and Payseur, 2012; Noor and Bennett, 2009; Renaut *et al*., 2013). Multiple recombinations could increase genetic differentiation and induce the formation of a new variety either by promoting gene flow or through the diversity‐reducing effects of linked selection. Indeed, the nature of recombination hotspots and the block‐like structure of linkage disequilibrium sites lead to substantial correlations of SNPs with many of their neighbours–certain SNPs are consistently tied to important agronomic traits and are inherited together. In any case, disentangling the relative importance of block inheritance and recombination in cotton artificial selection process remains a formidable challenge.

Here we re‐sequenced the allotetraploid genomes—*G. hirsutum* cultivar CRI‐12, its parents, and its progeny, along with 12 elite varieties with different genotypes to identify promising agronomic trait‐related genes in CRI‐12 by comparing genetic blocks and genetic variation among these varieties. The findings have broad implication for cotton breeding, marker‐assisted breeding and gene mapping. Besides, they are also potentially extendable to other crop plants.

## Results and discussion

### Genome resequencing, assembly and annotation

**Figure 1:**
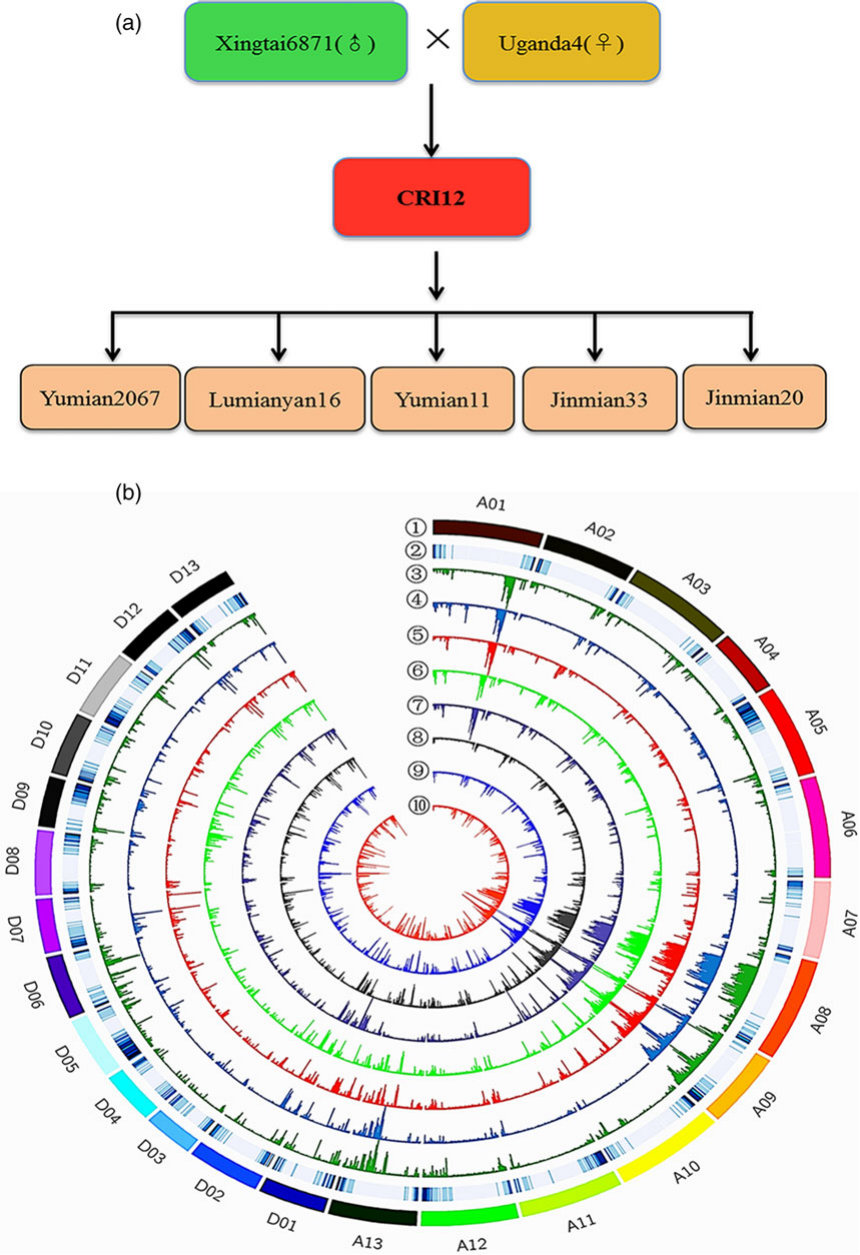
Genetic relationships and genome‐wide SNP variation analysis in eight Upland cottons. (a) among these eight accessions, CRI‐12 was the focal variety. Xingtai6871 comes from China and Uganda4 comes from Uganda. Yumian2067, Lumianyan16, Yumian11, Jinmian33 and Jinmian20 were formed using CRI‐12 as one of the two parents. Twelve elite varieties were also included in the accompanying analyses. (b) Upland cotton (*Gossypium hirsutum* L.) contains 26 chromosomes, including A subgenome (A01‐13) and D subgenome (D01‐13). The figure showed the distribution of whole‐genome wide variation on each chromosome. The outermost layer (①) represents chromosome names and other tracks, from the second outside circle (②) to inside, show gene density (window size = 1 Mb, sliding window = 1 Mb, nonoverlapping), Uganda4 (③, green), Xingtai6871 (④, blue), CRI‐12 (⑤, red), Yumian2067 (⑥, brilliant green), Lumianyan16 (⑦, blue grey), Yumian11 (⑧, grey), Jinmian33 (⑨, brilliant blue) and Jinmian20 (⑩, orange). The highest value of SNP, Indel and SV were 6000, 800 and 100, respectively. The deeper the colour is, the bigger the density.

CRI‐12, its parent cultivars, its five progeny cultivars (having CRI‐12 as a male or a female parent) and 12 elite cotton varieties were selected in the study (Figure 1a and Table S1). An allohaploid plant was derived from each variety and used for genome sequencing with a combined whole‐genome shotgun approach. Genome sequencing was performed using the Illumina HiSeq4000 platform with libraries having an insert size of approximately 500 bp. We generated a total of 1.2 Tb (30 × genome equivalent) of high‐quality paired‐end Illumina reads, which were preprocessed and filtered using SOAP2 (Table S2). Mapping rates were all above 99.9% and effective depths were above 98.50% in most varieties. High mapping rates and effective depths ensured that data were sufficiently reliable to proceed. After filtering out low‐quality reads, reliable data were used to identify both SNPs and small insertions‐deletions (indels) with SAM tools in an effort to define the amounts of structural variation present as well (Fu and Dooner, 2002; Swanson‐Wagner *et al*., 2010). We obtained 3 309 496 SNPs and 798 952 indels with average frequencies of 1 SNP per 813 bp and 1 indel per 2706 bp.

The genomic positions of these SNPs were examined in order to investigate their potential effects (Figure 1b). SNPs located in translation initiation sites,protein‐coding regions, translation termination sites and some other mis‐sense sites can potentially lead to changes in amino sequences, resulting in phenotypic alterations. SNP annotation indicated that approximately 91.48% were positioned in intergenic/intronic regions, while 8.32% were associated with protein‐coding regions (Table S3), including 55 734 nonsynonymous, 43 172 synonymous and 917 non‐sense mutations (Figure S1A). Large‐effect SNPs in two parents and CRI‐12 were showed in Figure S1B, some of which may change translation initiation sites and alternative splicing sites. We annotated 798 952 indels with the same method (Table S4), finding that nearly 76.46% indels were located in intergenic/intronic regions and 11.09% were found in coding regions, which are consistent with SNP annotation results. The presence of many repetitive sequences and transposons in *G. hirsutum* L. likely explains why most SNPs and indels are found in these noncoding regions (Li *et al*., 2015; Zhang *et al*., 2015). We calculated the number of Indels found in each of the CRI‐12 parent varieties (Uganda4 and Xingtai6871) and CRI‐12 itself (Figures S2 and S3), finding 74 682, 61 966 and 69 810 indels, respectively, with the lengths ranging from 5 bp to 50 860 bp; Table S5 and Figure S2.

Structural variations frequently tied to transposable element expansion and variations in repeat sequences have been previously found to positively correlate with genome size among maize varieties (Gopal, 1985; Kim *et al*., 2009; Oliveira *et al*., 1991). Accordingly, we investigated the structural variation present in each of the two CRI‐12 parents and CRI‐12, finally 46 145, 44 428 and 30 237 SVs (including insertions, deletions and others containing inversions, translocations, etc.) were identified in Uganda4, Xingtai6871 and CRI‐12, respectively. Among these variations, insertions found in Uganda4 and Xingtai6871 were far more than that in CRI‐12. Besides, 89.20% (26 971/30 237) of SVs were deletions. All these variants showed large changes of structural variations have taken place in CRI‐12 (Table S6 and Figure S4), which possibly explains why the cultivar CRI‐12 possesses improved agronomic traits compared to its parents.

### Artificial selection signals in leading morphotypes in elite cotton lines

**Figure 2:**
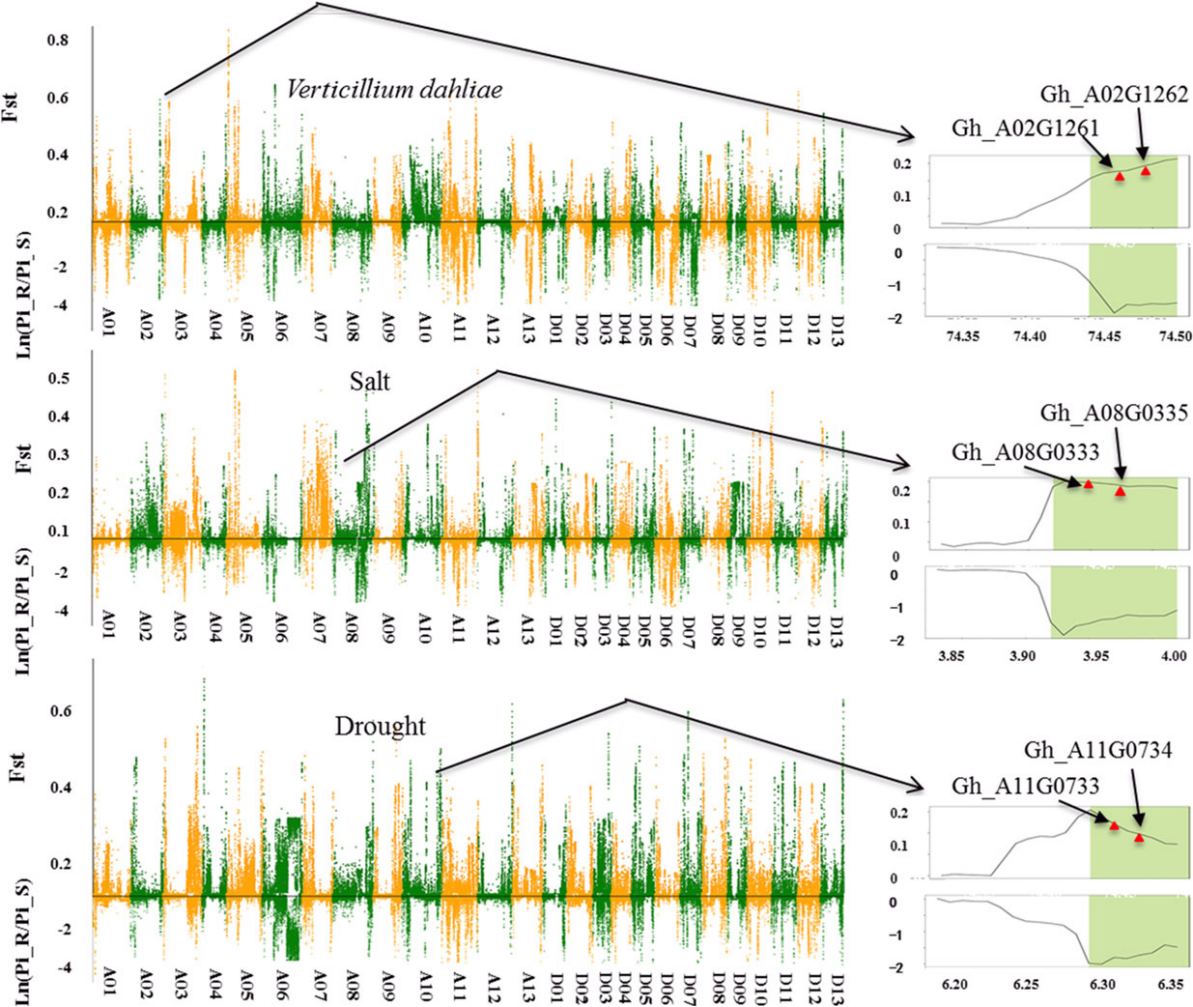
Whole‐genome selective sweep analysis for tolerance‐related genes in cotton. Genome‐wide distribution of *F*_st_ and ln (π_R/π_S) values (sliding window = 1 Mb). Orange and Green colours delineate 26 chromosomes in *Gossypium hirsutum* L. Magnified regions show two genes for each trait out of the three traits examined that showed strong selective signals.

Twenty well‐performing varieties with different specialties were clustered into three groups, each with two subgroups: tolerant/susceptible to *V. wilt* pathogenesis (*V. wilt* group), tolerant/sensitive to salt stress (salt group) and tolerant/sensitive to drought stress (drought group; Table S7), which were assessed using standard methods (Liu, 1989; Wang and Ma, 2002; Ye and Liu, 1998). Molecular identification of parents and progeny in the CRI‐12 family was conducted ([Link], [Link] and Figure S5). Using the SNP data obtained from resequencing varieties, we estimated the average pairwise diversity (π) and population differentiation statistics (*F*_ST_) between the sensitive and tolerant groups (Figures S6–S8). We screened selective sweep regions in the three trait groups by only including regions possessing outlying *F*_ST_ values and π ratios (<0.01, filtering more false positive genes; Figure 2). In total, we identified 420 genes across the three trait groups, with 118 in the *V. wilt* group, 126 in the salt group and 176 in the drought group (Table S10). The function and expression levels of these genes were obtained to assess their participation in agronomic trait improvement.

CRI‐12 was generated in a disease nursery of *V. wilt*, so high levels of disease tolerance, especially to *V. wilt* and *Fusarium oxysporum*, were the most significant characteristics in CRI‐12. We obtained 387 SNPs correlated with *V. wilt* and annotated 66 genes (Table S11), identifying a callose synthase, an ethanolaminephosphotransferase, a superoxide dismutase and a multidrug tolerance protein that differ in pairwise diversity and population differentiation between tolerant and susceptible *V. wilt* subgroups. Callose is a protective substance that cotton plants produce when attacked by pathogenic bacteria preventing bacteria from invading these callose‐producing cells and high expression of callose genes would be helpful for *V. wilt* resistance in plants (Li *et al*., 2009b; Manavella and Chan, 2009). Superoxide dismutase and ethanolaminephospho‐transferase both have been found associated with *V. wilt* tolerance, possibly allowing these select varieties to avoid *V. wilt* pathogenesis (Godon *et al*., 1993; Morales and Gottlieb, 1993). Domestication and artificial selection for one or a few valuable characteristics often reduces effective population size and genetic diversity (Gibbs *et al*., 2009; Ross‐Ibarra *et al*., 2007). Selective sweep analysis suggests these genes have been subjected to selection for their contributions to the improvement of disease tolerance in CRI‐12.

With resequencing data from 20 varieties, 65 genes were associated with the 373 salt‐group SNPs, while 86 genes were associated with the 587 drought‐group SNPs ([Link], [Link]). These genes were annotated and transcriptome data were obtained to investigate their functions. A remarkable proportion of genes were up‐regulated under salt (24.60%) and drought stress (38.37%), indicating their involvement in adaptation to these stressors. Genes related to transportin, aquaporin and hydrolase activity were highly correlated with abiotic stress. Furthermore, we discovered three genes (Gh_D08G2586, Gh_A08G1293 and Gh_A03G0274) involved in phyto‐hormone metabolism, of which one was correlated with signal transduction. Transcriptome analysis for these selected genes showed significant differences in expression, with all of these genes found in CRI‐12. These differences in expression may contribute to the phenotypic differences between these groups. We performed GO enrichment analysis for all identified genes using KEGG databases, the results showed that most genes were enriched for catalytic activity, cell and metabolic processes (Figures S9–S11).

### Inheritance and recombination of haplotype blocks caused by human selection in CRI‐12 family

**Figure 3:**
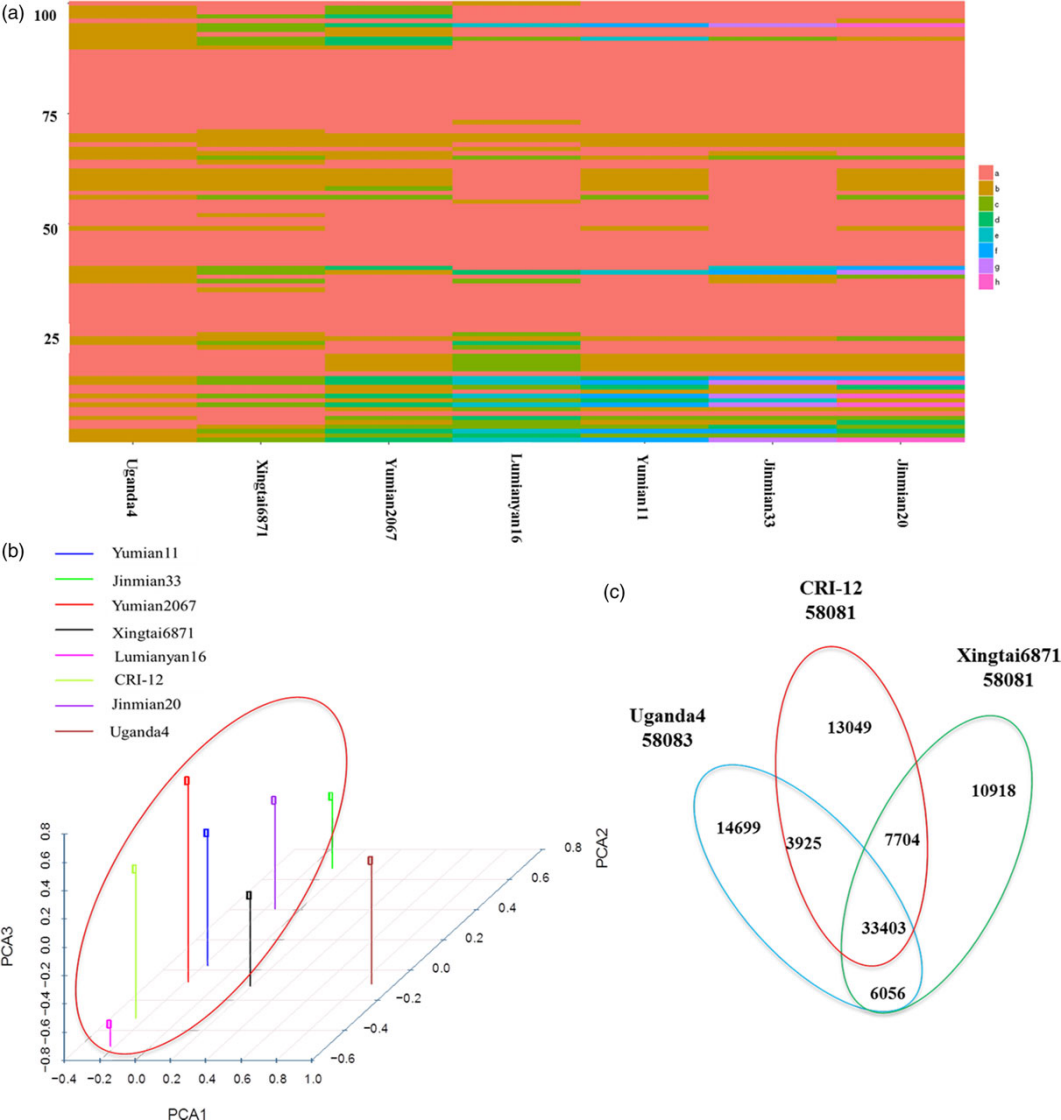
The distribution of SNP haplotype blocks in the eight CRI‐12 family varieties. (a) SNP haplotype blocks identified in CRI‐12 family varieties. Each line represents a SNP haplotype block, with the CRI‐12 family varieties listed along the *X* axis. When a haplotype block matched that of CRI‐12, we coloured it red (A). If it matched a second, different haplotype block, we applied another colour (B) and so on with the subsequent haplotypes and their associated colours (C, D, E, F, G, H). Lines may be different from each other. We found 31 894 SNP haplotype blocks to be shared among the CRI‐12 family of cultivars. Only 100 haplotype blocks with significant difference were shown here. (b) 3‐D principal component analysis. The horizontal axis represents principal component one and the two vertical axes represent the other two principal components. Different colours represent different cotton varieties. We divided these eight varieties into two groups. (c) A Venn diagram showing unique and shared SNP haplotype blocks in CRI‐12 and its two parents.

Several SNPs were linked together as a haplotype block within the initial 20 varieties, as well as within four sequenced *Gossypium* genomes, including *Gossypium raimondii*,* Gossypium arboreum*,* G. hirsutum* L. and *Gossypium barbadense*. We discovered 58 116 haplotype blocks across these varieties, with 23 752 haplotype blocks found to be shared by the eight varieties in the CRI‐12 family (including CRI‐12, two parents and five progeny) indicating that a high proportion (40.87%) of SNP haplotype blocks could be inherited from parents to progeny. These haplotype blocks were found with the lengths ranging from 50 bp to 201 178 bp with an average length of 6352 ± 70 bp (Figure 3a and Table S14). These SNP haplotype blocks were unequally distributed across the 26 allotetraploid cotton chromosomes, the Dt subgenome retaining more than half (63.62%) of haplotype blocks and the At subgenome retaining only 36.38% of SNP haplotype blocks, indicating that the Dt subgenome may be more easily inherited. Annotation of these genes found within inherited haplotype blocks showed that most genes were related to large/small subunit ribosomal proteins (Gh_A03G1453 and Gh_D13G2138, related to photosynthesis), chromosome transmission fidelity proteins and DNA polymerase zeta subunit (Gh_D08G0292 and Gh_D08G0296, related to DNA replication), cellulose synthases (Haigler *et al*., 2012) and mitogen‐activated protein kinases (Gh_D12G0590 and Gh_D12G1351, related to cell proliferation). These genes may be indispensable for normal cotton growth. Furthermore, we also have identified genes closely related to fibre length and strength, including a phosphoenolpyruvate carboxylase gene (PEPC; Brosche *et al*., 2014), Gh_D10G0347 (a RING/U‐box superfamily protein) and Gh_D09G1417 (a leucine‐rich repeat protein; Zhang *et al*., 2015). In total, we obtained eight and five haplotype blocks, containing ten and six genes correlated with high fibre yield and fibre quality, respectively. Given that these characters subject to artificial selection before the CRI‐12 family became a variety, we should expect that fibre yield‐ and quality‐related genes would be found in each of its member cultivars.

To investigate the inheritance within the CRI‐12 family, we calculated the number of haplotype blocks in each parent (Uganda4 or Xingtai6871), which were inherited to CRI‐12 and its five progeny, filtering out haplotype blocks shared by the two parents. We found that approximately 4.22% (1003/23 752) of Uganda4 haplotype blocks and 14.40% (3420/23 752) of Xingtai6871 haplotype blocks were shared across the CRI‐12 family ([Link], [Link]). The greater haplotype block contribution by Xingtai6871 is likely due to its being a domestic variety and CRI‐12 progeny likely having domestic co‐parentage. Principal component analysis (PCA; Figure 3b) also indicated that the genetic background of Uganda4 was relatively different from the other seven varieties in the CRI‐12 family. These results seem to indicate that SNP haplotype blocks can be inherited from parental varieties and passed on to progeny to varying degrees dependent on the genetic relationships had with each of the progeny contributing parental varieties. The results may also be explained by the existence of different artificial selection pressures placed on these contributing lines. At any rate, these data allow us to propose why some cotton materials with one or few sought‐after characteristics were chosen to breed new varieties in the long‐term domestication and artificial selection of cotton.

In order to investigate whether these inherited haplotype blocks were correlated with the traits examined in this study, we singled out those haplotype blocks that included genes identified in the selective sweep approach. The 420 genes obtained from the selective sweep were used to identify 24 haplotype blocks, including 2, 2 and 20 haplotype blocks correlated with *V. wilt*, salt‐ and drought‐ tolerance, respectively (Table S17). Furthermore, haplotype block length is correlated with the total number of genes found in a given block, larger blocks containing more genes. Among the haplotype blocks, we found that 22 blocks were located in the Dt subgenome, suggesting that haplotype blocks in Dt subgenome may be closely related to abiotic stress tolerances, which is consistent with the hypothesis that PSGs (positively selected genes) in Dt subgenome facilitate cotton development by regulating abiotic stress tolerance (Zhang *et al*., 2015). Many superior characters found in CRI‐12 were also found in its five progeny (Table S7), which supports this conclusion. The salt‐ and drought‐ tolerance of cotton have improved greatly in recent decades as a result of massive breeding efforts. The improvements in phenotypic traits are reflected in the genomes of these cultivars with their whole‐genome polymorphisms providing us the means to identify signatures of artificial selection from cotton breeding (Huang *et al*., 2012; Hufford *et al*., 2012; Morrell *et al*., 2012).

**Figure 4:**
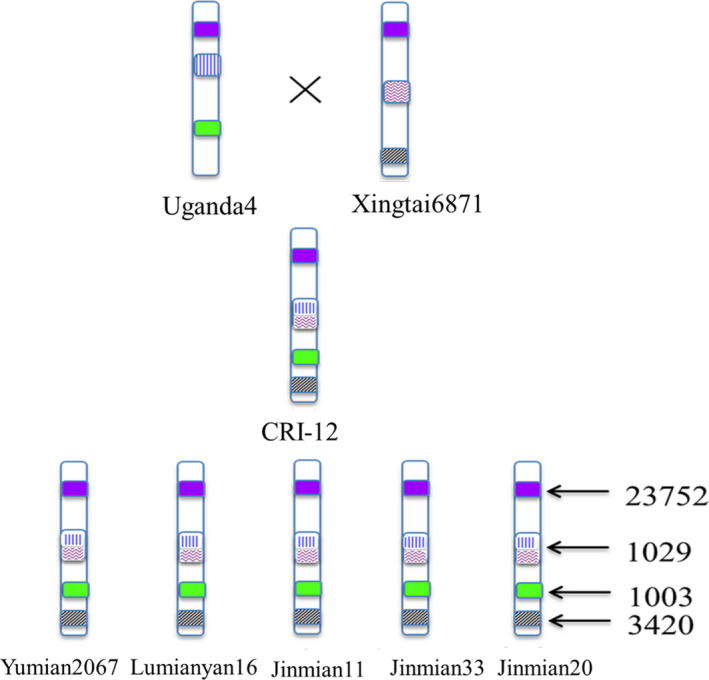
A schematic diagram of haplotype blocks inheritance and recombination in CRI‐12 family. The haplotype blocks in Uganda4 and Xingtai6871 could be recombined into one haplotype in CRI‐12, and these recombined haplotype blocks in CRI‐12 were inherited to its progeny (Yumian2067, Lumianyan16, Yumian11, Jinmian33 and Jinmian20). Purple modules represent haplotype blocks shared by eight varieties. Blue modules represent haplotype blocks inherited only Uganda4 and black modules represent haplotype blocks inherited from Xingtai6871. The recombined modules represent haplotype blocks recombined from Uganda4 and Xingtai6871. Each type of haplotype blocks were listed once in the figure. Not all haplotypes are visualized here. The numbers 23 752, 1029, 1003 and 3420 represent sums of each type of haplotype blocks.

Additionally, tremendous amounts of recombination were discovered among the eight cotton varieties using their SNP haplotype blocks. We determined SNP haplotype blocks which were shared by and restricted to Uganda4, Xingtai6871 and CRI‐12 and its progeny. In total, it appears that 14 699 haplotype blocks in Uganda4 and 10 918 haplotype blocks in Xingtai6871 recombined to only 1029 haplotype blocks in CRI‐12, which were then inherited to five progeny (Figures 3c and 4 and Table S18). These haplotype blocks were nearly equally distributed across the At and Dt subgenomes with an average length of 13 345 ± 594 bp. Other plant studies also showed that a large amount of historic recombination has occurred in Z. *mays* throughout the genome (Tenaillon *et al*., 2001; Wright, 2005). All these results could contribute to our interpretation of the breeding mechanism and the model of phenotypic inheritance in cotton. This recombination is likely important to the formation and improvement of new important traits and deserves further investigation. Recombination and inheritance of haplotype blocks including a subset of artificial selection loci may contribute to the development of phenotypes of agronomic importance.

### Haplotype block polymorphism between different genomes suggested their evolution and divergence

**Figure 5:**
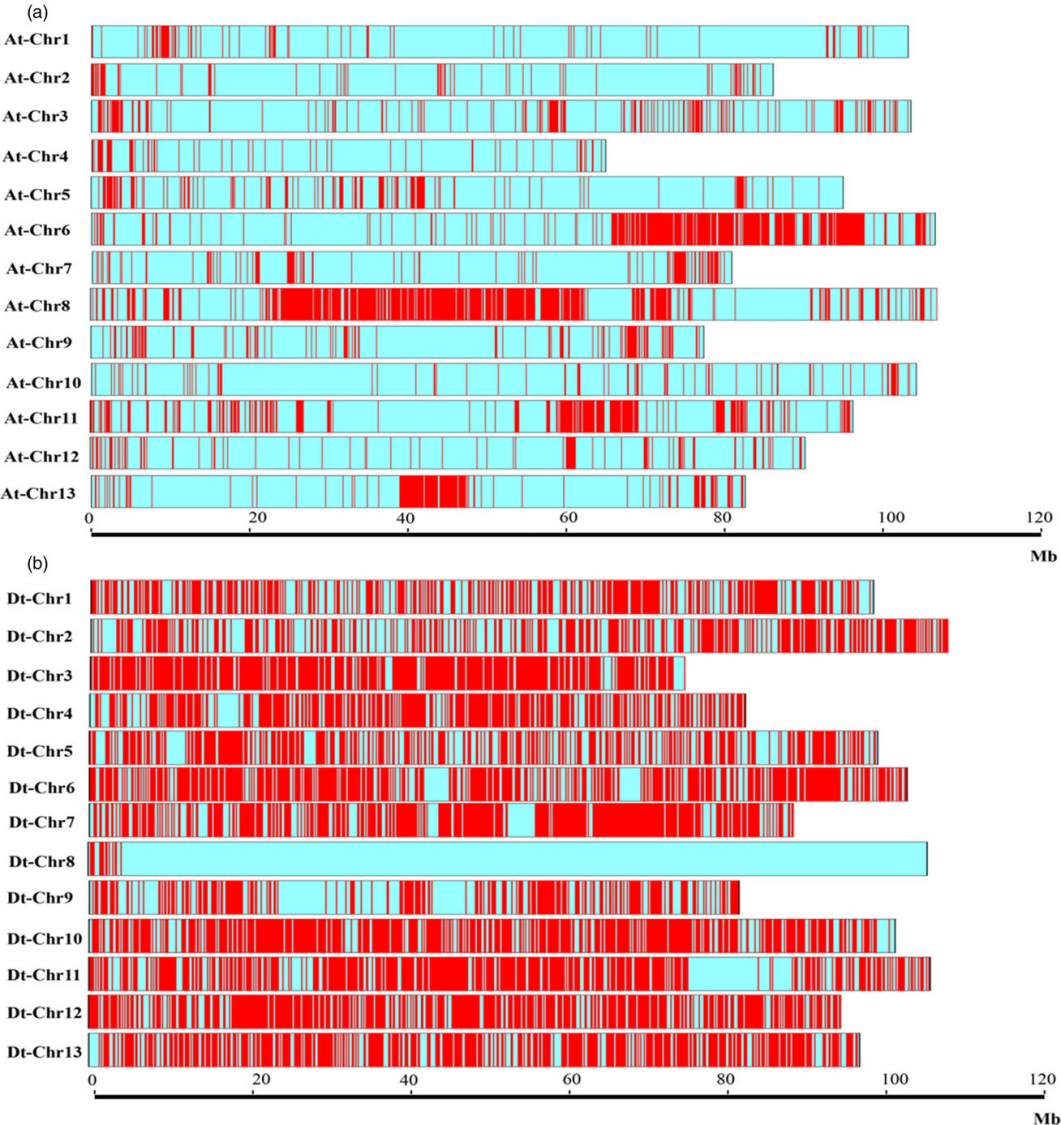
The distribution of haplotypes shared by eight cotton varieties in At and Dt subgenome. (a) represents the distribution of haplotypes shared by eight cotton varieties in At subgenome; (b) represents the distribution of haplotypes shared by eight cotton varieties in Dt subgenome. Red bars mean haplotype density, more red bars, higher the haplotype density.

We compared SNP haplotype block polymorphisms among the genomes of *G. raimondii*,* G. arboreum*,* G. hirsutum* L. and *G. barbadense* to uncover the haplotype blocks shared between and distinct to diploids and tetraploids with similar methods and criteria. In total, 56 275 haplotype blocks were obtained, accounting for 39.97% of the A genome, while only 33 haplotype blocks were found to be shared between the two diploids, suggesting that the differentiation between *G. arboreum* and *G. raimondii* have begun since their initial divergence. Previous studies showed *G. arboreum* could be cultivated, whereas *G. raimondii* produces no spinnable fibre (Li *et al*., 2014), and the results related to the large differences in haplotype blocks between the *G. arboreum* (A‐genome) and the *G. raimondii* (D‐genome) possibly explained this problem. Similarly we identified 55 077 haplotype blocks between *G. hirsutum* L. and *G. barbadense* and only 1894 haplotype blocks were discovered to be shared. Huge difference between *G. hirsutum* L. and *G. barbadense* suggested that these two functional genomes are vastly different. Besides, a high proportion of haplotype blocks were found in the Dt subgenome (61.18%; Figure 5), which possibly explained the origin of haplotype blocks containing genes of high *V. wilt*‐, salt‐ and drought‐ tolerance were from *G. raimondii* (D genome, a wild diploid cotton species, identified with *V. wilt*‐, salt‐ and drought‐ tolerance) when undergoing domestication. These results also were helpful to investigate subgenome evolution and the functions of a cluster of linked genes in one block.

Reference genome TM‐1, the genetic standard of allotetraploid *G. hirsutum*, was also used to identify haplotype blocks along with the heretofore mentioned 20 cotton varieties, providing valuable information for functional research and explaining why CRI‐12 and its five progeny have so many merits. The comparison between TM‐1, and CRI‐12 and five progeny showed that 18 526 haplotype blocks differed between the two groups. Functional annotation analysis of genes located in these differential haplotype blocks showed that genes linked to responses to stimuli (700), catalytic activity (1697), metabolic processes (1992), single‐organism processes (1597) and regulation of macromolecular complexes were enriched. These results were consistent with the fact that significant differences in *V. wilt*‐, salt‐ and drought‐ tolerance were found between CRI‐12 and TM‐1.

Based on above analysis, CRI‐12–the most widely grown in China and in the world, bred through the hybridization of the domestic cotton variety Xingtai6871 (♂) and the distant Uganda4 (♀)–once accounted for more than half of the nation's growing area of cotton for its adaptation and disease tolerances in addition to its capacity to produce large yields of high‐quality fibre. At present, CRI‐12 has been partially substituted by some new and more insect‐resistant varieties. Twenty varieties were clustered into three groups and used to conduct selective sweep analyses to identify *V. wilt*‐, salt‐ and drought‐ tolerance genes to explain why CRI‐12 demonstrates high fibre‐yield, good fibre‐quality and extensive adaptation. We also conducted the molecular identification of parents and progeny in the CRI‐12 family and identified 420 genes related to *V. wilt*, salt and drought tolerance by selective sweep analysis, revealing the genetic mechanism of artificial selection of cotton breeding. This method has been implemented to identify traits‐related genes in plants and other systems (Hazzouri *et al*., 2015; Qi *et al*., 2013; Yang *et al*., 2016) and can be applied to a broader array of selected traits to provide insights into underlying physiological mechanisms. Using 20 single plants to represent 20 highly homozygous varieties, we demonstrated that SNP haplotype blocks with important variation correlated with adversity‐tolerance traits could be recombined and inherited in the breeding process of CRI‐12 family, providing an important clue for future crop breeding in cotton and other crops.

Generally speaking, larger blocks screened, more genes inherited. Genomic regions of valuable functional genes with lower recombination frequency usually were inherited together. The larger the blocks, more genes would be contained. Although haplotype block inheritance has been proposed and reported in other systems (Altshuler *et al*., 2005; Chia *et al*., 2012; Jia *et al*., 2013), the mechanisms of their inheritance and recombination in cotton pedigree have not been previously observed. Additionally, the potential association between haplotype block inheritance and trait improvement suggest that cotton and other crop‐breeding programs might benefit from focusing their efforts on haplotype blocks containing a subset of valuable genes, especially those affecting *V. wilt*, salt and drought tolerances. Our large dataset of loci bearing signatures of recent selection provides a foundation for further genetic improvement in cotton. Ideally, approaches incorporating SNP haplotype blocks are to be implemented into future crop improvement efforts. Developing a haplotype block selection system would be an effective approach towards enhancing artificial selection, allowing future studies to develop improved insight into the mechanisms involved in inheritance.

Taken together, based on analyses of resequencing of *G. hirsutum* L. *cv* CRI‐12, its parental cultivars and progeny cultivars, and transcriptome‐wide analyses of gene expression and functional annotations, 3.3 million SNPs were identified and 118, 126 and 176 genes were found correlating with three biotic and abiotic tolerances in CRI‐12, respectively. On the basis of these data, we found haplotype block inheritance correlated with many agronomically important genes under artificial selection. So we develop a haplotype block selection system and this would be an effective approach towards enhancing artificial selection and this work would guide breeding efforts for the genetic improvement of cotton for breeders.

## Materials and methods

### Plant materials and sequencing

We used CRI‐12, which is high‐yielding [the cotton lint yield before frost were 17.5% and 11.5% higher than the control variety in the Yellow river (Jinmian7) and Yangtze river (86‐1) regions], high‐quality (29.9 mm fibre length, 5855 m/g fineness and 3.91 g of single fibre tensile strength) and stronger multidisease tolerance (14.3 of *V. wilt* index and 4.1 of *F*. *wilt* index); its parents Xingtai6871 (♂) and Uganda4 (♀); and its progeny (having CRI‐12 as one of its parents) Yumian2067, Lumianyan16, Yumian11, Jinmian33 and Jinmian20 for DNA sequencing. The cotton seeds were preserved through many successive generations of self‐fertilization in the mid‐term repository of cotton germplasm at the Institute of Cotton Research, Chinese Academy of Agricultural Sciences. Twelve elite cotton varieties with different traits (mainly salt, drought and disease tolerance) including Zhong9807, CRI35, Kanghuangwei164, GK50, Xinyan96‐48, Zhongs9612, Zhongzhimian2, Jimian11, Zhong571, Shuomian3, Chuangmian47 and Lumian378, were selected for DNA or RNA isolation and high‐throughput sequencing (Table S7). Seeds with uniform sizes were selected and grown in sand in the greenhouses at the Institute of Cotton Research of the Chinese Academy of Agricultural Sciences (ICR, CAAS). One representative plant for each variety was chosen for DNA isolation at approximately three weeks old, using the two youngest leaves for the isolation.

DNA libraries were constructed following the manufacturer's instructions (Illumina Genome Analyzer, San Diego, CA) and paired‐end reads were generated using the Illumina Hiseq4000 service provided by Bio‐technology Corporation 1 GENE (Hangzhou, China). Paired‐end sequencing libraries with insert sizes of 500 bp were prepared using an Illumina paired‐end library kit. Using a whole genome shotgun strategy, 30 × coverage raw data of 150 bp paired‐end reads was generated. All reads were preprocessed and filtered using the software SOAP 2 before mapping on the NBI *G. hirsutum* genome. Any reads with any of the following characteristics were removed: (i) ≥5% unidentified nucleotides; (ii) >10 nt aligned to the adapter sequence, allowing ≤10% mismatches; (iii) >50% bases having phred quality of <5; (iv) possession of putative PCR duplicates generated by PCR amplification in the library construction process. High‐quality reads were retained for the further analyses.

### Alignment, SNP calling, indel calling and genotyping

Paired‐end reads (clean reads) were mapped against the NBI *G*. *hirsutum* genome using BWA (ver.0.7.10) program (Li and Durbin, 2010) with parameters: ‘bwa mem ‐M ‐t 8 ‐k 32 –R’. SAM tools (Li *et al*., 2009a) were then used to convert mapping results to bam format and sort them from name order into coordinate order. In order to reduce the number of miscalls of Indels, we realigned raw gapped alignment with the GATK toolkit (ver.3.2‐2; McKenna *et al*., 2010). Then we used mpileup to produce a BCF file that contained all of the locations in the genome. We used this information to call genotypes and reduced our list of sites to those found to be variants by passing this file into Bcftools call. Finally, we filtered data using commands as follows: ‘Bcftools filter ‐G 10 ‐g 10 ‐O z ‐o output ‐s LOWQUAL ‐i “%QUAL>=30 && MQ >=30 && DP >=5” input’.

SNPs and Indels were then divided from filtered data using the shell command. 3 309 496 SNPs and 798 952 Indels were detected in total. There were about one SNP per 831 bases and one Indel per 2706 bases. The identified SNPs and indels were further annotated with ANNOVAR tool (Wang *et al*., 2010). On the basis of the detected SNPs, we obtained genotypes with in‐house perl scripts. According to the annotations of SNPs identified and the definition of gene elements (exons, introns and UTR regions; Chung *et al*., 2006), then we positioned these SNPs in order to investigate their functions.

### Structure variation

According to the mapping result, a remarkable difference between the gap information and the insert size of paired‐end reads was observed, which is usually indicative of candidate structural variants (SVs). Genome‐wide detection of SVs based on sam format data was achieved with the software SOAPsv (Version 5.4.0) which can predict insertions, deletions, etc.

### Principal component analysis

Principal component analysis of the population was performed using the software EIGENSOFT (v3.0; http://genepath.med.harvard.edu/~reich/Software.htm; Patterson *et al*., 2006). To perform PCA on the individuals, SNPs of 20 individuals (including eight accessions of CRI‐12 family members and 12 varieties to serve as references for *V. wilt*‐, salt‐ and drought‐ tolerance analysis) were used after filtering with Bcftools (http://www.htslib.org/doc/bcftools-1.0.html). The first two principal components were used to visualize the genetic relatedness among individuals and investigated groups. We plotted the PCA analysis image using R package 3.2.

### Identification of candidate genes under selection

The allele frequencies of variable sites were used in two complementary approaches to identify regions potentially affected by long‐term selection (Lam *et al*., 2010). We calculated population fixation statistics (*F*_ST_) and nucleotide diversity (θ_π_) for each sliding window (in 100 kb windows with 10 kb step size). *F*_ST_, which calculates the genomic differentiation for two groups, was used to evaluate the tolerance of genomic differentiation on candidates between two groups (Akey *et al*., 2002). θ_π_ measures the genomic diversity and was used to estimate the genomic diversity of these varieties (Tajima, 1983).

*F*_ST_ is defined as:

$$F_{\mathrm{st}}=\frac{\pi_{\mathrm{Between}}-\pi_{\mathrm{Within}}}{\pi_{\mathrm{Between}}}$$

where π_Between_ and π_Within_ represent the average number of pairwise differences between two individuals sampled from different populations (π_Between_) or the same population (π_Within_).

ln (θπ_R/θπ_S) is defined as:

$$\ln\left(\theta \pi \_R/\theta \pi \_S\right)=\ln\left(\frac{\text{Resistance subgroup}\;\theta \pi }{\text{Sensitive subgroup}\;\theta \pi }\right)$$

where θπ_R and θπ_S represent the tolerance subgroup θπ and sensitive subgroup θπ, respectively.

We calculated the log value of θ_π_ ratios. The putative selection targets were designated as the top 1% of log‐odds ratios for both θ_π_ and *F*_ST_. Both θ_π_ and *F*_ST_ were calculated using PopGen package of Bioperl (ver. 1.6.923). We compared the resistant subgroup and sensitive subgroup and thus obtained candidate genes. We totally investigated genome regions for genes that have undergone a selective sweep for *V. wilt*, salt and drought tolerance and sensitivity.

### Gene functional annotation

Candidate genes were annotated within functional categories based on Gene Ontology (Ashburner *et al*., 2000) and KEGG (Kanehisa and Goto, 2000) databases using in house perl scripts. The genes were annotated by a homology‐based method. Transcriptome data and gene expression analysis of *V. wilt*, salt and drought could refer to previous data of Shao *et al*. (Shao *et al*., 2015), our salt‐transcriptome data (relative data was unpublished) and drought‐transcriptome data (Lu *et al*., 2016) in our laboratory. And analysis method used, threshold value set and accession numbers of raw data were all could be found in corresponding researches.

### Haplotype block

A haplotype (haploid genotype) is a group of genes in an organism that are inherited together from a single parent. Haplotype block is a specific arrangement of adjacent alleles in a given region of genomic DNA that is inherited as a ‘block’, probably because its recombination frequency is lower than in other parts of the genome. In practice, a haplotype block is characterized by a series of single nucleotide polymorphisms (SNPs) in linkage disequilibrium. The history of recombination between a pair of SNPs can be estimated with the use of the normalized measure of allelic association, D’. Using the definition given by Gabriel (Gabriel *et al*., 2002), the pairs were considered in ‘strong LD’ if the one‐sided upper 95% confidence boundary of D’ was 0.98 (i.e. consistent with no historical recombination) and the lower boundary was above 0.7. If the D’ for a pair of SNPs was lower than 0.7, the next haplotype block began. That is to say, only nearby SNPs with continuous combinations were included in a haplotype block, and few SNPs having low LD with adjacent markers were omitted in the haplotype‐based association study. The haplotype blocks were detected by the software Haploview (Barrett *et al*., 2005). Comparing the blocks between CRI‐12 and other cotton samples, we found the shared blocks and annotated the genes function within the blocks region. We also used the previous transcriptome data (Lu *et al*., 2016) to validate the function of candidate genes.

## Author contributions

W.W.Y., J.Z.Y. and X.K.L. conceived and designed the experiments. X.K.L., X.Q.F., D.L.W, C.W.S, J.Y.W. and X.G.C. conducted the experiment and led the data analysis. D.L.W., M.R.H., J.J.W., S.W. and W.L.F performed the field data survey. X.G.C., C.W.S., L.X.G., Z.J.Y., X.G.W. and J.P. contributed to sample preparations, literature search, DNA sequencing and data analysis. R.F.C., N.S., B.L.Z, K.A.G., M.G.H., X.J.Z and M.M. contributed to data analysis and manuscript editing. X.K.L., X.Q.F. D.L.W, J.Z.Y. and W.W.Y. wrote and revised the manuscript. All data have been deposited in Bioproject and the accession number is PRJNA374751.

## Competing financial interests

The authors declare no competing financial interests.

## Supporting information


**Figure S1** Functional classification of SNPs in protein‐coding regions.
**Figure S2** Genome‐wide indel variation analysis in eight upland cottons.
**Figure S3** The distribution of indels with different lengths in the CDS region.
**Figure S4** Genome‐wide structural variation (SV) analysis in eight upland cottons.
**Figure S5** Molecular identification of the relationship among eight cotton varieties by SSR.
**Figure S6** Selection signals associated with *Verticillium dahlia*.
**Figure S7** Selection signals associated with salt stress.
**Figure S8** Selection signals associated with drought stress.
**Figure S9** Pathway analysis of *Verticillium dahliae* related genes identified.
**Figure S10** Pathway analysis of salt related genes identified.
**Figure S11** Pathway analysis of drought related genes identified.

**Table S1** Cotton varieties used in the study.

**Table S2** Statistics of sequence data.

**Table S3** Annotation of SNPs detected.

**Table S4** Annotation of indels discovered.

**Table S5** Statistics of indels between two parents and CRI‐12.

**Table S6** Statistics of structural variations (SV) between two parents and CRI‐12.

**Table S7** Groups of cotton accessions.

**Table S8** Molecular identification of parent‐offspring in CRI‐12 family.

**Table S9** Primers used for molecular identification.

**Table S10** Genes identified by selective sweep analysis.

**Table S11** Annotation of SNPs related with *Verticillium wilt*.

**Table S12** Annotation of salt‐related SNPs.

**Table S13** Annotation of drought‐related SNPs.

**Table S14** SNP modules polymorphism analysis in CRI12 family.

**Table S15** SNP haplotypes shared by Uganda4 and CRI‐12 and 5 descendants.

**Table S16** SNP haplotypes shared by Xingtai6871 and CRI‐12 and 5 descendants.

**Table S17** Inherited SNP haplotypes were correlated with plant resistance.

**Table S18** Haplotypes recombined and inherited in CRI‐12 family.

## References

[pbi13030-bib-0001] Akey, J.M. , Zhang, G. , Zhang, K. , Jin, L. and Shriver, M.D. (2002) Interrogating a high‐density SNP map for signatures of natural selection. Genome Res. 12, 1805–1814. 12466284 10.1101/gr.631202 PMC187574

[pbi13030-bib-0002] Altshuler, D. , Brooks, L.D. , Chakravarti, A. , Collins, F.S. , Daly, M.J. , Donnelly, P. , Gibbs, R.A. *et al*. (2005) A haplotype map of the human genome. Nature, 437, 1299–1320. 16255080 10.1038/nature04226 PMC1880871

[pbi13030-bib-0003] Ashburner, M. , Ball, C.A. , Blake, J.A. , Botstein, D. , Butler, H. , Cherry, J.M. , Davis, A.P. *et al*. (2000) Gene ontology: tool for the unification of biology. Nat. Genet. 25, 25–29. 10802651 10.1038/75556 PMC3037419

[pbi13030-bib-0004] Barrett, J.C. , Fry, B. , Maller, J. and Daly, M.J. (2005) Haploview: analysis and visualization of LD and haplotype maps. Bioinformatics, 21, 263–265. 15297300 10.1093/bioinformatics/bth457

[pbi13030-bib-0006] Brosche, M. , Blomster, T. , Salojarvi, J. , Cui, F.Q. , Sipari, N. , Leppala, J. , Lamminmaki, A. *et al*. (2014) Transcriptomics and functional genomics of ROS‐induced cell death regulation by RADICAL‐INDUCED CELL DEATH1. PLoS Genet. 10, e1004112. 24550736 10.1371/journal.pgen.1004112 PMC3923667

[pbi13030-bib-0007] Chia, J.M. , Song, C. , Bradbury, P.J. , Costich, D. , de Leon, N. , Doebley, J. , Elshire, R.J. *et al*. (2012) Maize HapMap2 identifies extant variation from a genome in flux. Nat. Genet. 44, 803–807. 22660545 10.1038/ng.2313

[pbi13030-bib-0008] Chung, B.Y. , Simons, C. , Firth, A.E. , Brown, C.M. and Hellens, R.P. (2006) Effect of 5′UTR introns on gene expression in *Arabidopsis thaliana* . BMC Genom. 7, 120. 10.1186/1471-2164-7-120 PMC1482700 16712733

[pbi13030-bib-0009] Fu, H.H. and Dooner, H.K. (2002) Intraspecific violation of genetic colinearity and its implications in maize. Proc. Natl Acad. Sci. USA, 99, 9573–9578. 12060715 10.1073/pnas.132259199 PMC123182

[pbi13030-bib-0010] Gabriel, S.B. , Schaffner, S.F. , Nguyen, H. , Moore, J.M. , Roy, J. , Blumenstiel, B. , Higgins, J. *et al*. (2002) The structure of haplotype blocks in the human genome. Science, 296, 2225–2229. 12029063 10.1126/science.1069424

[pbi13030-bib-0011] Gibbs, R.A. , Taylor, J.F. , Van Tassell, C.P. , Barendse, W. , Eversole, K.A. , Gill, C.A. , Green, R.D. *et al*. (2009) Genome‐wide survey of SNP variation uncovers the genetic structure of cattle breeds. Science, 324, 528–532. 19390050 10.1126/science.1167936 PMC2735092

[pbi13030-bib-0012] Godon, C. , Caboche, M. and Daniel‐Vedele, F. (1993) Transient plant gene expression: a simple and reproducible method based on flowing particle gun. Biochimie, 75, 591–595. 8268259 10.1016/0300-9084(93)90065-z

[pbi13030-bib-0013] Gopal, T.V. (1985) Gene transfer method for transient gene expression, stable transformation, and cotransformation of suspension cell cultures. Mol. Cell. Biol. 5, 1188–1190. 2987679 10.1128/mcb.5.5.1188-1190.1985 PMC366838

[pbi13030-bib-0014] Haigler, C.H. , Betancur, L. , Stiff, M.R. and Tuttle, J.R. (2012) Cotton fiber: a powerful single‐cell model for cell wall and cellulose research. Front. Plant Sci. 3, 104. 22661979 10.3389/fpls.2012.00104 PMC3356883

[pbi13030-bib-0015] Hazzouri, K.M. , Flowers, J.M. , Visser, H.J. , Khierallah, H.S.M. , Rosas, U. , Pham, G.M. , Meyer, R.S. *et al*. (2015) Whole genome re‐sequencing of date palms yields insights into diversification of a fruit tree crop. Nat. Commun. 6, 8824. 26549859 10.1038/ncomms9824 PMC4667612

[pbi13030-bib-0016] Huang, X.H. , Kurata, N. , Wei, X.H. , Wang, Z.X. , Wang, A. , Zhao, Q. , Zhao, Y. *et al*. (2012) A map of rice genome variation reveals the origin of cultivated rice. Nature, 490, 497–503. 23034647 10.1038/nature11532 PMC7518720

[pbi13030-bib-0017] Hufford, M.B. , Xu, X. , van Heerwaarden, J. , Pyhajarvi, T. , Chia, J.M. , Cartwright, R.A. , Elshire, R.J. *et al*. (2012) Comparative population genomics of maize domestication and improvement. Nat. Genet. 44, 808–811. 22660546 10.1038/ng.2309 PMC5531767

[pbi13030-bib-0018] Jia, Y.L. , Jia, M.H. , Wang, X.Y. and Liu, G.J. (2012) Indica and Japonica crosses resulting in linkage block and recombination suppression on rice chromosome 12. PLoS ONE, 7, e43066. 22912788 10.1371/journal.pone.0043066 PMC3422337

[pbi13030-bib-0019] Jia, G.Q. , Huang, X.H. , Zhi, H. , Zhao, Y. , Zhao, Q. , Li, W.J. , Chai, Y. *et al*. (2013) A haplotype map of genomic variations and genome‐wide association studies of agronomic traits in foxtail millet (*Setaria italica*). Nat. Genet. 45, 957–961. 23793027 10.1038/ng.2673

[pbi13030-bib-0020] Jiang, C.X. , Wright, R.J. , El‐Zik, K.M. and Paterson, A.H. (1998) Polyploid formation created unique avenues for response to selection in Gossypium (cotton). Proc. Natl Acad. Sci. USA, 95, 4419–4424. 9539752 10.1073/pnas.95.8.4419 PMC22504

[pbi13030-bib-0021] Kanehisa, M. and Goto, S. (2000) KEGG: kyoto encyclopedia of genes and genomes. Nucleic Acids Res. 28, 27–30. 10592173 10.1093/nar/28.1.27 PMC102409

[pbi13030-bib-0022] Kim, M.J. , Baek, K. and Park, C.M. (2009) Optimization of conditions for transient Agrobacterium‐mediated gene expression assays in Arabidopsis. Plant Cell Rep. 28, 1159–1167. 19484242 10.1007/s00299-009-0717-z

[pbi13030-bib-0023] Lai, J.S. , Li, R.Q. , Xu, X. , Jin, W.W. , Xu, M.L. , Zhao, H.N. , Xiang, Z.K. *et al*. (2010) Genome‐wide patterns of genetic variation among elite maize inbred lines. Nat. Genet. 42, 1027–1030. 20972441 10.1038/ng.684

[pbi13030-bib-0024] Lam, H.M. , Xu, X. , Liu, X. , Chen, W. , Yang, G. , Wong, F.L. , Li, M.W. *et al*. (2010) Resequencing of 31 wild and cultivated soybean genomes identifies patterns of genetic diversity and selection. Nat. Genet. 42, 1053–1059. 21076406 10.1038/ng.715

[pbi13030-bib-0025] Li, H. and Durbin, R. (2010) Fast and accurate long‐read alignment with Burrows‐Wheeler transform. Bioinformatics, 26, 589–595. 20080505 10.1093/bioinformatics/btp698 PMC2828108

[pbi13030-bib-0026] Li, H. , Handsaker, B. , Wysoker, A. , Fennell, T. , Ruan, J. , Homer, N. , Marth, G. *et al*. (2009a) The sequence alignment/map format and SAMtools. Bioinformatics, 25, 2078–2079. 19505943 10.1093/bioinformatics/btp352 PMC2723002

[pbi13030-bib-0027] Li, J.F. , Park, E. , von Arnim, A.G. and Nebenfuhr, A. (2009b) The FAST technique: a simplified Agrobacterium‐based transformation method for transient gene expression analysis in seedlings of *Arabidopsis* and other plant species. Plant Methods, 5, 6. 19457242 10.1186/1746-4811-5-6 PMC2693113

[pbi13030-bib-0028] Li, F.G. , Fan, G.Y. , Wang, K.B. , Sun, F.M. , Yuan, Y.L. , Song, G.L. , Li, Q. *et al*. (2014) Genome sequence of the cultivated cotton *Gossypium arboreum* . Nat. Genet. 46, 567–572. 24836287 10.1038/ng.2987

[pbi13030-bib-0029] Li, F.G. , Fan, G.Y. , Lu, C.R. , Xiao, G.H. , Zou, C.S. , Kohel, R.J. , Ma, Z.Y. *et al*. (2015) Genome sequence of cultivated upland cotton (*Gossypium hirsutum* TM‐1) provides insights into genome evolution. Nat. Biotechnol. 33, 524–530. 25893780 10.1038/nbt.3208

[pbi13030-bib-0030] Liu, J.D. (1989) Identification and application on drought‐tolerance appraisal of cotton germplasm resources. China Cotton, 4, 12.

[pbi13030-bib-0031] Lu, X.K. , Chen, X.G. , Mu, M. , Wang, J.J. , Wang, X.G. , Wang, D.L. , Yin, Z.J. *et al*. (2016) Genome‐wide analysis of long noncoding RNAs and their responses to drought stress in cotton (*Gossypium hirsutum* L.). PLoS ONE, 11, e0156723. 27294517 10.1371/journal.pone.0156723 PMC4905672

[pbi13030-bib-0032] Luikart, G. , England, P.R. , Tallmon, D. , Jordan, S. and Taberlet, P. (2003) The power and promise of population genomics: from genotyping to genome typing. Nat. Rev. Genet. 4, 981–994. 14631358 10.1038/nrg1226

[pbi13030-bib-0033] Manavella, P.A. and Chan, R.L. (2009) Transient transformation of sunflower leaf discs via an Agrobacterium‐mediated method: applications for gene expression and silencing studies. Nat. Protoc. 4, 1699–1707. 19876029 10.1038/nprot.2009.178

[pbi13030-bib-0034] McKenna, A. , Hanna, M. , Banks, E. , Sivachenko, A. , Cibulskis, K. , Kernytsky, A. , Garimella, K. *et al*. (2010) The genome analysis toolkit: a MapReduce framework for analyzing next‐generation DNA sequencing data. Genome Res. 20, 1297–1303. 20644199 10.1101/gr.107524.110 PMC2928508

[pbi13030-bib-0035] Morales, M.J. and Gottlieb, D.I. (1993) A polymerase chain reaction‐based method for detection and quantification of reporter gene expression in transient transfection assays. Anal. Biochem. 210, 188–194. 8489016 10.1006/abio.1993.1171

[pbi13030-bib-0036] Morrell, P.L. , Buckler, E.S. and Ross‐Ibarra, J. (2012) Crop genomics: advances and applications. Nat. Rev. Genet. 13, 85–96. 10.1038/nrg3097 22207165

[pbi13030-bib-0037] Nachman, M.W. and Payseur, B.A. (2012) Recombination rate variation and speciation: theoretical predictions and empirical results from rabbits and mice. Philos. Trans. R. Soc. Lond. B Biol. Sci. 367, 409–421. 22201170 10.1098/rstb.2011.0249 PMC3233716

[pbi13030-bib-0038] Noor, M.A. and Bennett, S.M. (2009) Islands of speciation or mirages in the desert? Examining the role of restricted recombination in maintaining species. Heredity (Edinb), 103, 439–444. 19920849 10.1038/hdy.2009.151 PMC2809014

[pbi13030-bib-0039] Oliveira, M.M. , Barroso, J. and Pais, M.S. (1991) Direct gene transfer into *Actinidia deliciosa* protoplasts: analysis of transient expression of the CAT gene using TLC autoradiography and a GC‐MS‐based method. Plant Mol. Biol. 17, 235–242. 1863775 10.1007/BF00039498

[pbi13030-bib-0040] Paterson, A.H. , Wendel, J.F. , Gundlach, H. , Guo, H. , Jenkins, J. , Jin, D.C. , Llewellyn, D. *et al*. (2012) Repeated polyploidization of Gossypium genomes and the evolution of spinnable cotton fibres. Nature, 492, 423–427. 23257886 10.1038/nature11798

[pbi13030-bib-0041] Patterson, N. , Price, A.L. and Reich, D. (2006) Population structure and eigenanalysis. PLoS Genet. 2, 2074–2093. 10.1371/journal.pgen.0020190 PMC1713260 17194218

[pbi13030-bib-0042] Qi, J.J. , Liu, X. , Shen, D. , Miao, H. , Xie, B.Y. , Li, X.X. , Zeng, P. *et al*. (2013) A genomic variation map provides insights into the genetic basis of cucumber domestication and diversity. Nat. Genet. 45, 1510–1515. 24141363 10.1038/ng.2801

[pbi13030-bib-0043] Renaut, S. , Grassa, C.J. , Yeaman, S. , Moyers, B.T. , Lai, Z. , Kane, N.C. , Bowers, J.E. *et al*. (2013) Genomic islands of divergence are not affected by geography of speciation in sunflowers. Nat. Commun. 4, 1827. 23652015 10.1038/ncomms2833

[pbi13030-bib-0044] Ross‐Ibarra, J. , Morrell, P.L. and Gaut, B.S. (2007) Plant domestication, a unique opportunity to identify the genetic basis of adaptation. Proc. Natl Acad. Sci. USA, 104(Suppl 1), 8641–8648. 17494757 10.1073/pnas.0700643104 PMC1876441

[pbi13030-bib-0045] Shao, B.X. , Zhao, Y.L. , Chen, W. , Wang, H.M. , Guo, Z.J. , Gong, H.Y. , Sang, X.H. *et al*. (2015) Analysis of upland cotton (*Gossypium hirsutum*) response to *Verticillium dahliae* inoculation by transcriptome sequencing. Genet. Mol. Res. 14, 13120–13130. 26535625 10.4238/2015.October.26.8

[pbi13030-bib-0046] Swanson‐Wagner, R.A. , Eichten, S.R. , Kumari, S. , Tiffin, P. , Stein, J.C. , Ware, D. and Springer, N.M. (2010) Pervasive gene content variation and copy number variation in maize and its undomesticated progenitor. Genome Res. 20, 1689–1699. 21036921 10.1101/gr.109165.110 PMC2989995

[pbi13030-bib-0047] Tajima, F. (1983) Evolutionary relationship of DNA sequences in finite populations. Genetics, 105, 437–460. 6628982 10.1093/genetics/105.2.437 PMC1202167

[pbi13030-bib-0048] Tan, L.W. and Liu, Z.D. (1992) Researches on selection and varietal traits of Zhongmian12. Sci. Agri. Sinica 23, 12–19.

[pbi13030-bib-0049] Tenaillon, M.I. , Sawkins, M.C. , Long, A.D. , Gaut, R.L. , Doebley, J.F. and Gaut, B.S. (2001) Patterns of DNA sequence polymorphism along chromosome 1 of maize (*Zea mays ssp mays* L.). Proc. Natl Acad. Sci. USA, 98, 9161–9166. 11470895 10.1073/pnas.151244298 PMC55390

[pbi13030-bib-0050] Wang, X.F. and Ma, Z.Y. (2002) A new method for identification of cotton *Verticillium wilt* resistance. Cotton Sci. 14, 231–233.

[pbi13030-bib-0051] Wang, K. , Li, M. and Hakonarson, H. (2010) ANNOVAR: functional annotation of genetic variants from high‐throughput sequencing data. Nucleic Acids Res. 38, e164. 20601685 10.1093/nar/gkq603 PMC2938201

[pbi13030-bib-0052] Wang, K.B. , Wang, Z.W. , Li, F.G. , Ye, W.W. , Wang, J.Y. , Song, G.L. , Yue, Z. *et al*. (2012) The draft genome of a diploid cotton *Gossypium raimondii* . Nat. Genet. 44, 1098–1104. 22922876 10.1038/ng.2371

[pbi13030-bib-0053] Wang, Y.Q. , Hao, C.Y. , Zheng, J. , Ge, H.M. , Zhou, Y. , Ma, Z.Q. and Zhang, X.Y. (2015) A haplotype block associated with thousand‐kernel weight on chromosome 5DS in common wheat (*Triticum aestivum* L.). J. Integr. Plant Biol. 57, 662–672. 25318826 10.1111/jipb.12294

[pbi13030-bib-0054] Wendel, J.F. (1989) New world tetraploid cottons contain old world cytoplasm. Proc. Natl Acad. Sci. USA, 86, 4132–4136. 16594050 10.1073/pnas.86.11.4132 PMC287403

[pbi13030-bib-0055] Wright, S.I. (2005) The effects of artificial selection on the maize genome (vol 308, pg 1310, 2005). Science, 310, 54. 10.1126/science.1107891 15919994

[pbi13030-bib-0056] Yang, J.H. , Liu, D.Y. , Wang, X.W. , Ji, C.M. , Cheng, F. , Liu, B.N. , Hu, Z.Y. *et al*. (2016) The genome sequence of allopolyploid *Brassica juncea* and analysis of differential homoeolog gene expression influencing selection. Nat. Genet. 48, 1225–1232. 27595476 10.1038/ng.3657

[pbi13030-bib-0057] Ye, W.W. and Liu, J.D. (1998) Technique and application on salt‐tolerance appraisal of cotton germplasm resources. China Cotton, 25, 34–38.

[pbi13030-bib-0058] Yuan, D.J. , Tang, Z.H. , Wang, M.J. , Gao, W.H. , Tu, L.L. , Jin, X. , Chen, L.L. *et al*. (2015) The genome sequence of Sea‐Island cotton (*Gossypium barbadense*) provides insights into the allopolyploidization and development of superior spinnable fibres. Sci. Rep. 5, 17662. 26634818 10.1038/srep17662 PMC4669482

[pbi13030-bib-0059] Zhang, T.Z. , Hu, Y. , Jiang, W.K. , Fang, L. , Guan, X.Y. , Chen, J.D. , Zhang, J.B. *et al*. (2015) Sequencing of allotetraploid cotton (*Gossypium hirsutum* L. acc. TM‐1) provides a resource for fiber improvement. Nat. Biotechnol. 33, 531–537. 25893781 10.1038/nbt.3207

