# Supplementary material for: Resequencing of cv CRI‐12 family reveals haplotype block inheritance and recombination of agronomically important genes in artificial selection
Source: Plant Biotechnol J. 2018 Dec 3;17(5):945–55. doi: 10.1111/pbi.13030 (PMC6587942; doi:10.1111/pbi.13030)
Supplement: Supplementary file 1 — Figure S1 Functional classification of SNPs in protein‐coding regions. Figure S2 Genome‐wide indel variation analysis in eight upland cottons. Figure S3 The distribution of indels with different lengths in the CDS region. Figure S4 Genome‐wide structural variation (SV) analysis in eight upland cottons. Figure S5 Molecular identification of the relationship among eight cotton varieties by SSR. Figure S6 Selection signals associated with Verticillium dahlia. Figure S7 Selection signals associated with salt stress. Figure S8 Selection signals associated with drought stress. Figure S9 Pathway analysis of Verticillium dahliae related genes identified. Figure S10 Pathway analysis of salt related genes identified. Figure S11 Pathway analysis of drought related genes identified. [file PBI-17-945-s001.pdf]

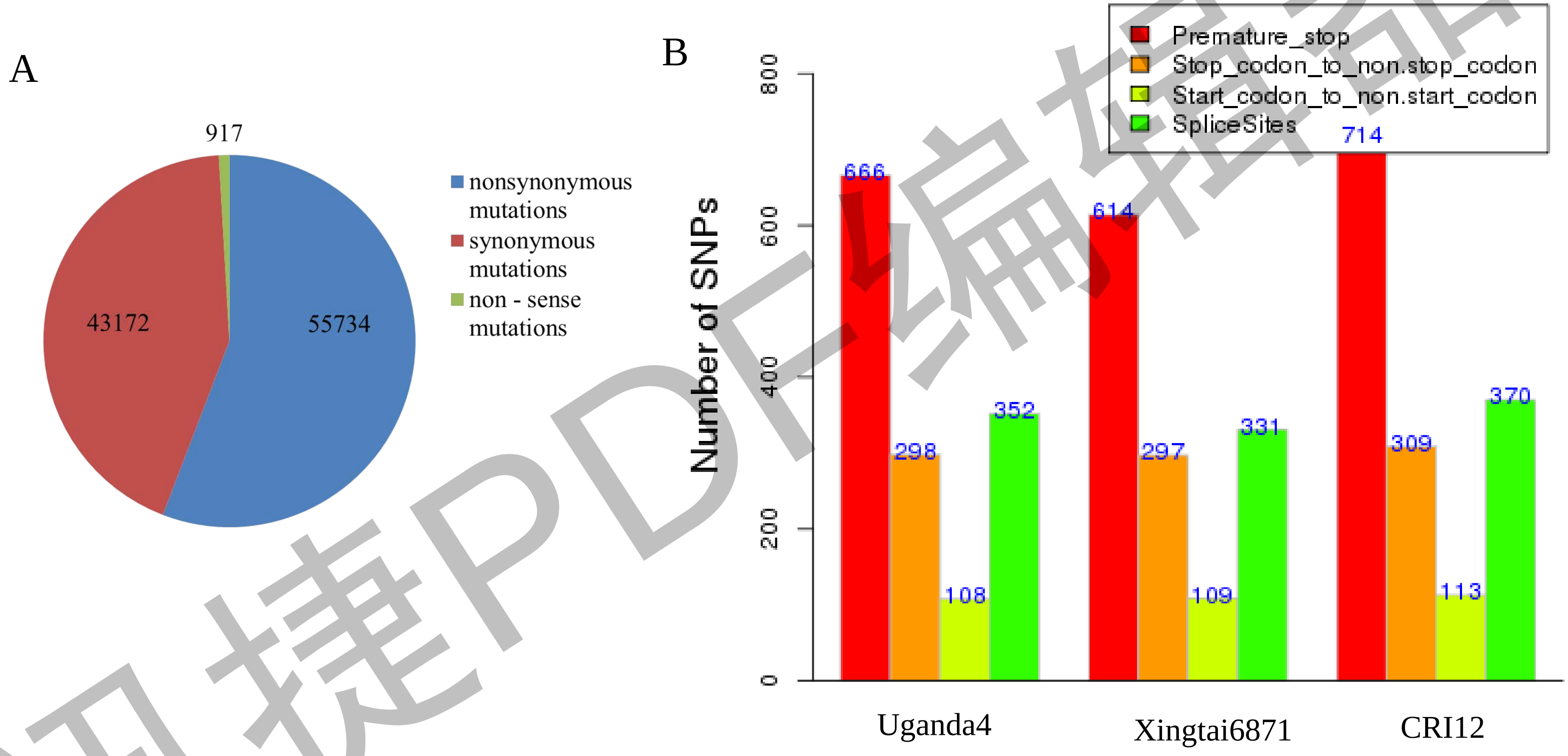

Supplementary figure 1 Functional classification of SNPs in protein-coding regions

A, Percentage of SNP in protein-coding regions; B, Number of large-effect SNPs.

迅捷PDF编辑器

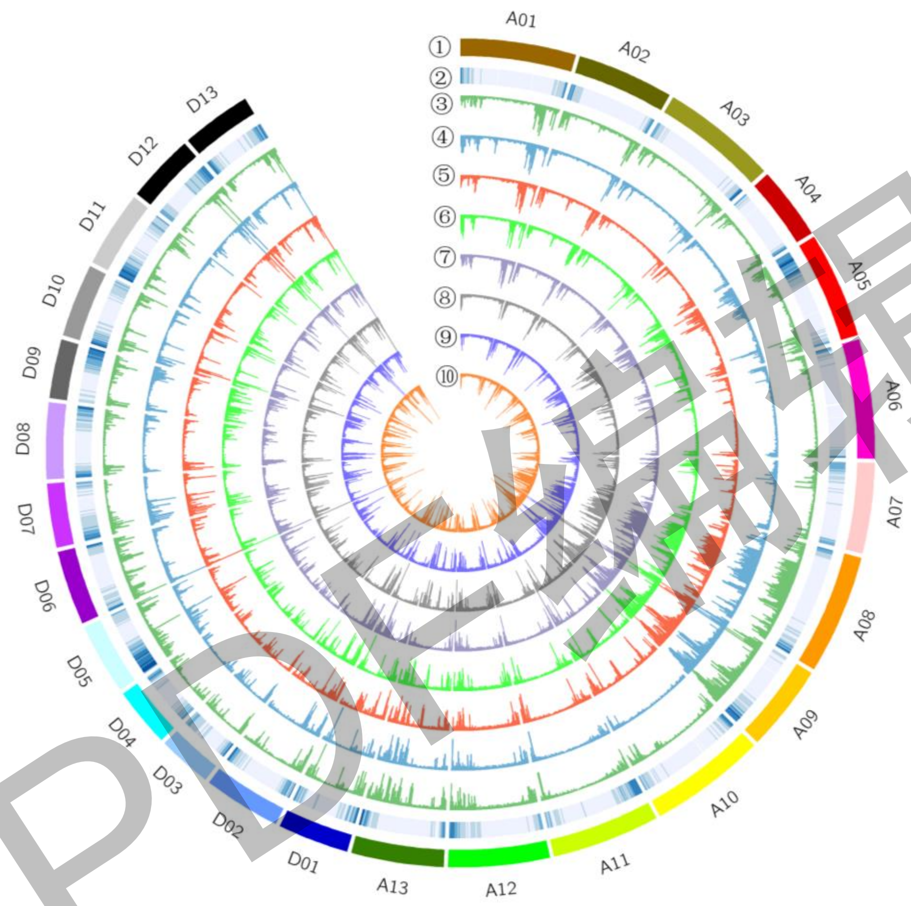

Supplementary Figure 2 Genome-wide indel variation analysis in 8 upland cottons.

Upland cotton (*Gossypium hirsutum* L.) contains 26 chromosomes,including the A subgenome (A01-13) and the D subgenome (D01-13). The figure shows the distribution of indels on each chromosome. The outermost layer (①) displays chromosome names, the second circle from the outsided (②) shows gene density (window size =1Mb, sliding window=1Mb, non-overlapping), and the remaining layers display indel distributions for Uganda4 (③,green), Xingtai6871 (④, blue), CRI-12 (⑤,red), Yumian2067 (⑥, brilliant green), Lumianyan16 (⑦, blue grey), Yumian11 (⑧, grey), Jinmian33 (⑨, brilliant blue), and Jinmian20 (⑩, orange). The length of indels ranged from 5 bp to 50,860 bp. Indels located on scaffolds were not visualized.

迅捷PDF编辑器

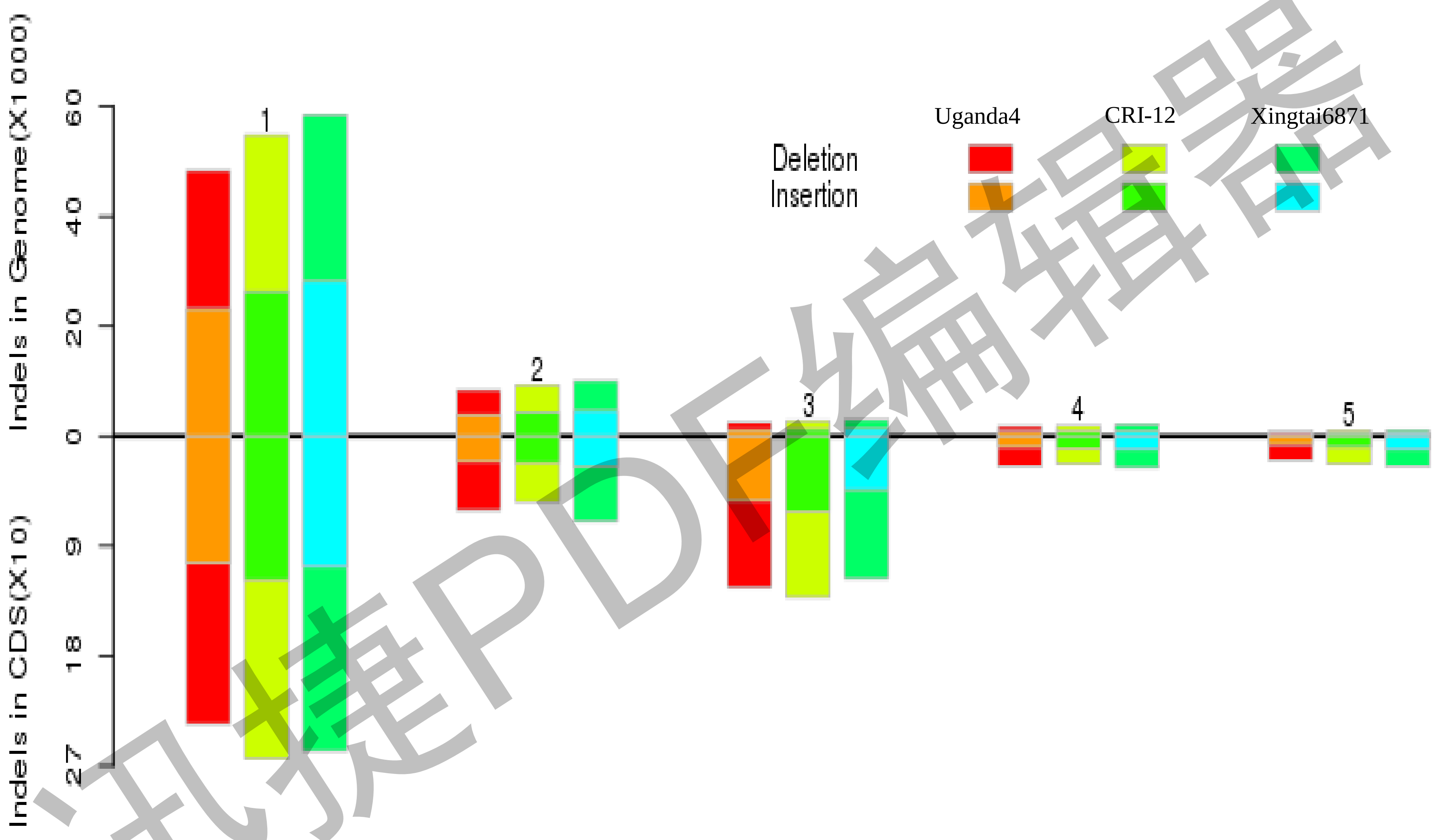

Supplementary Figure 3 The distribution of indels with different lengths in the CDS region

迅捷PDF编辑器

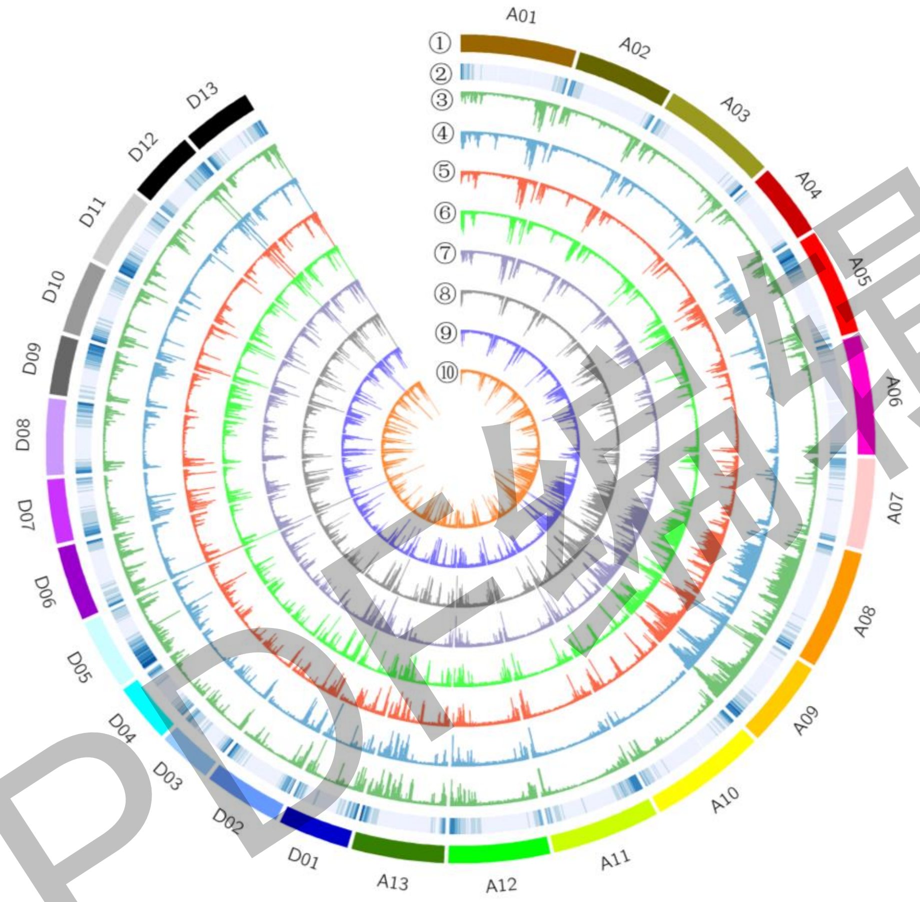

Supplementary Figure 4 Genome-wide structual variation (SV) analysis in 8 upland cottons

Upland cotton (*Gossypium hirsutum* L.) contains 26 chromosomes, including the A subgenome (A01-13) and the D subgenome (D01-13). The figure shows the distribution of SVs on each chromosome. The outermost layer (①) displays chromosome names, the second (②) shows gene density (sliding window=1Mb, non-overlapping-darker color indicates greater gene density), and the remaining layers display SV distributions for Uganda4 (③,green), Xingtai6871 (④, blue), CRI-12 (⑤,red), Yumian2067 (⑥, brilliant green), Lumianyan16 (⑦, blue grey), Yumian11 (⑧, grey), Jinmian33 (⑨, brilliant blue), and Jinmian20 (⑩, orange). SVs located on scaffolds are not visualized.

迅捷PDF编辑器

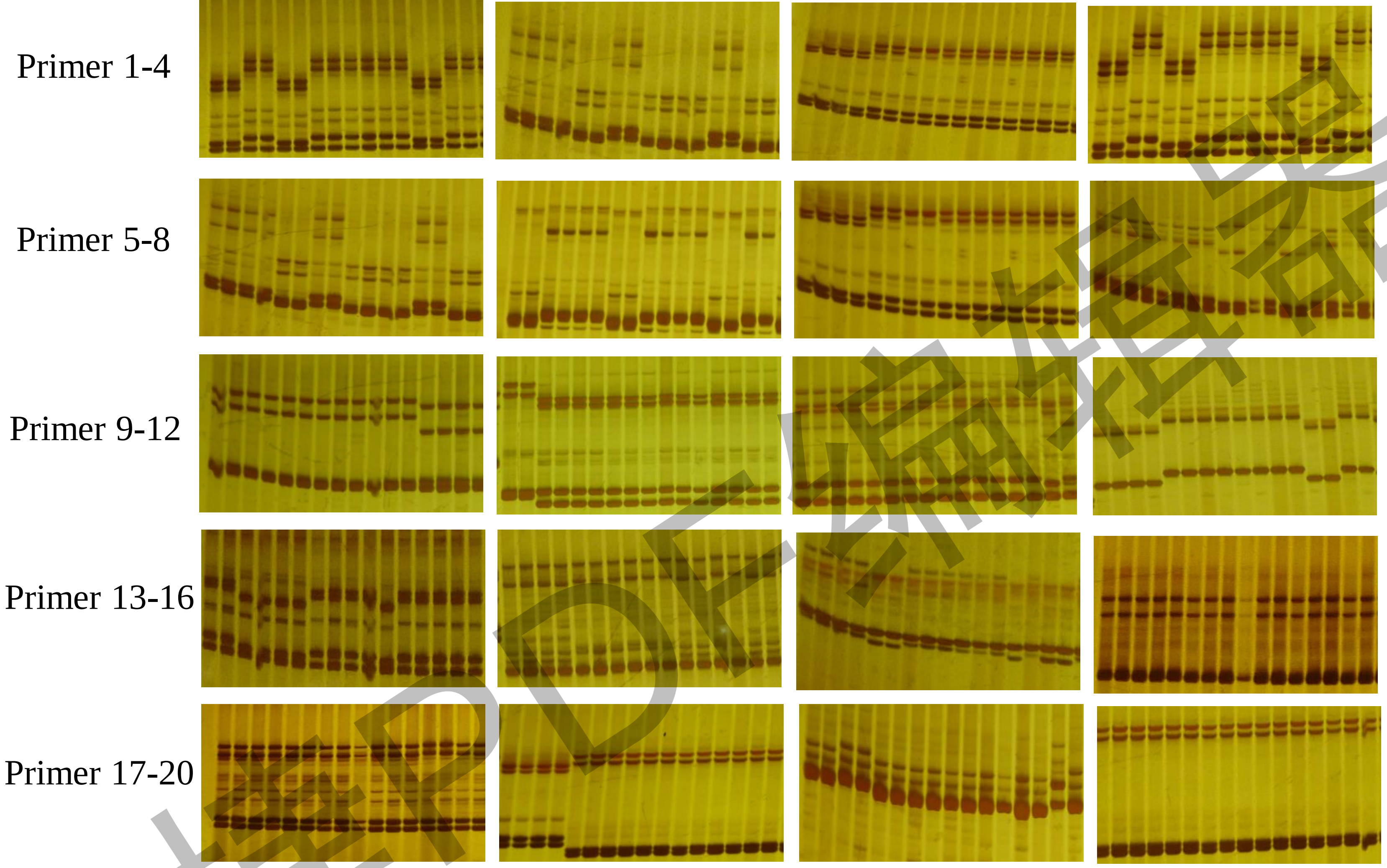

Supplementary Figure 5 Molecular identification of the relationship among 8 cotton varieties by SSR

We selected 20 primers to validate the relationships among the eight CRI-12 family cotton varieties with SSR technology after line purity had been ascertained for each variety. Two plants were selected from each variety. Primer sequences could be found in Supplementary Table 17.

迅捷PDF编辑器

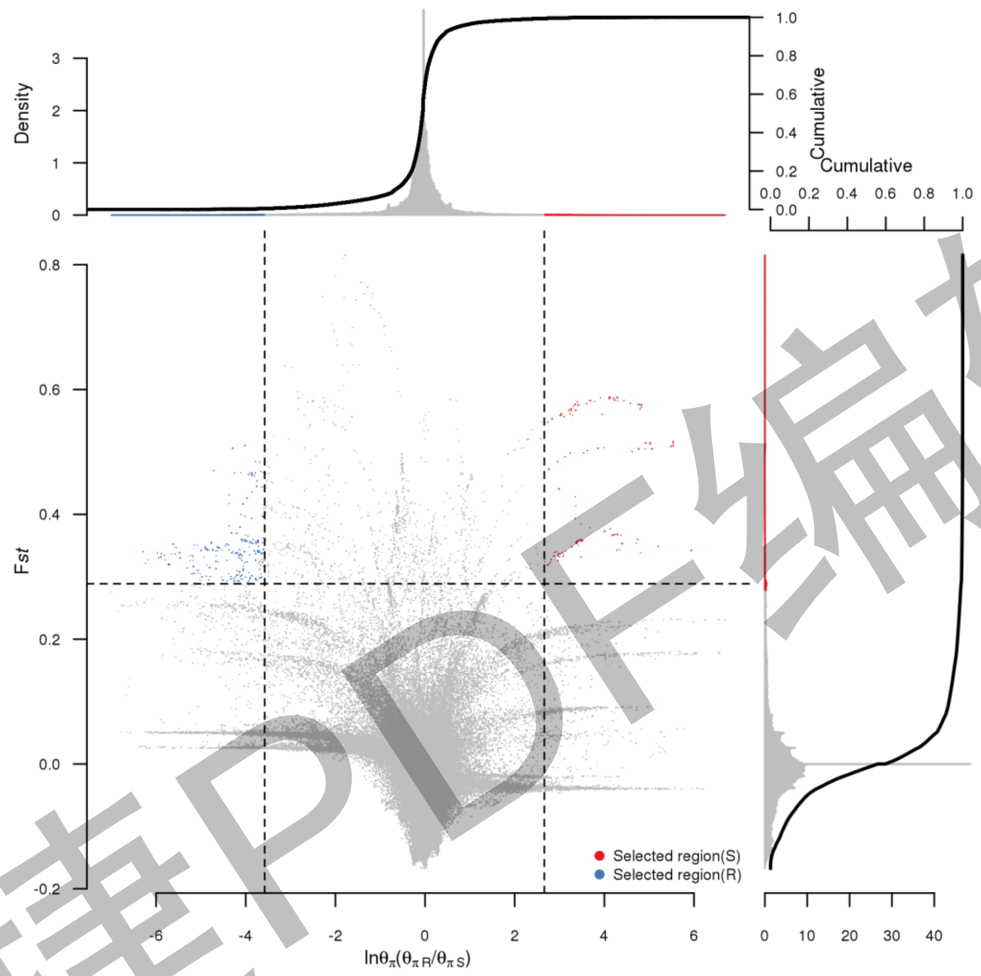

Supplementary Figure 6 Selection signals associated with *Verticillium dahliae*

迅捷PDF编辑器

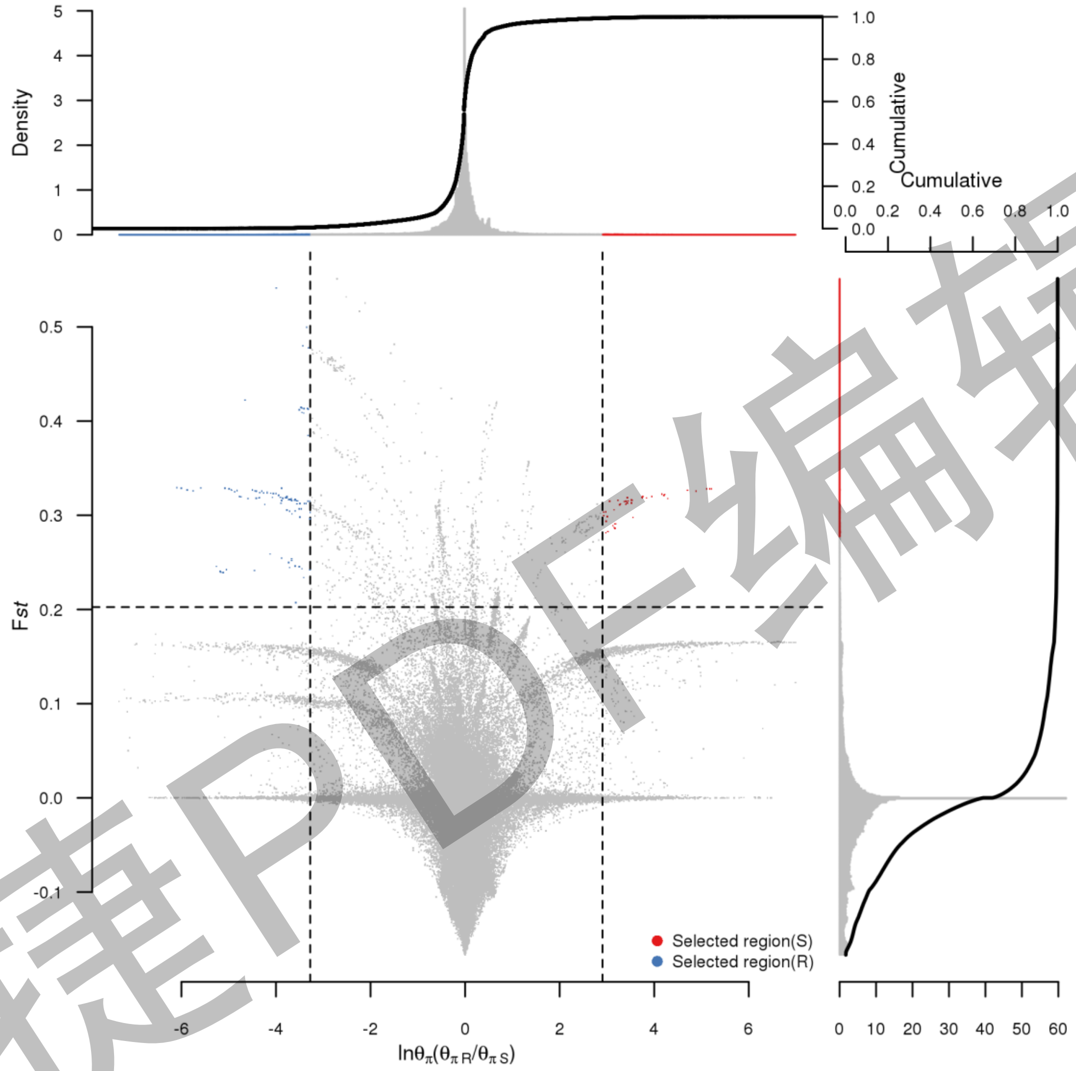

Supplementary Figure 7 Selection signals associated with salt stress

迅捷PDF编辑器

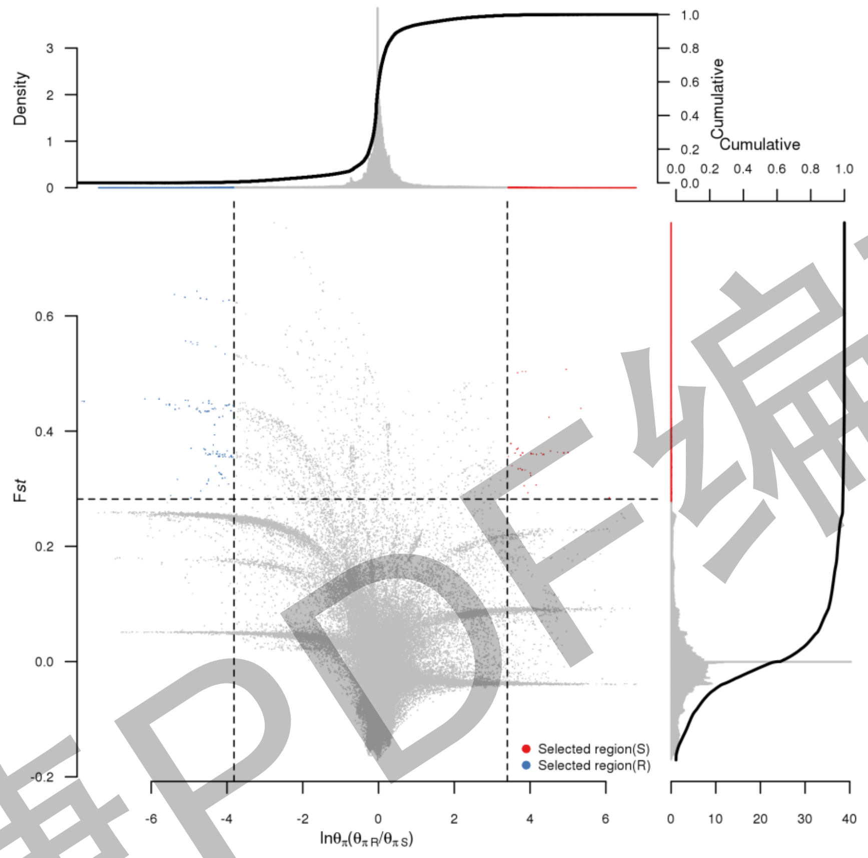

Supplementary Figure 8 Selection signals associated with drought stress

迅捷PDF编辑器

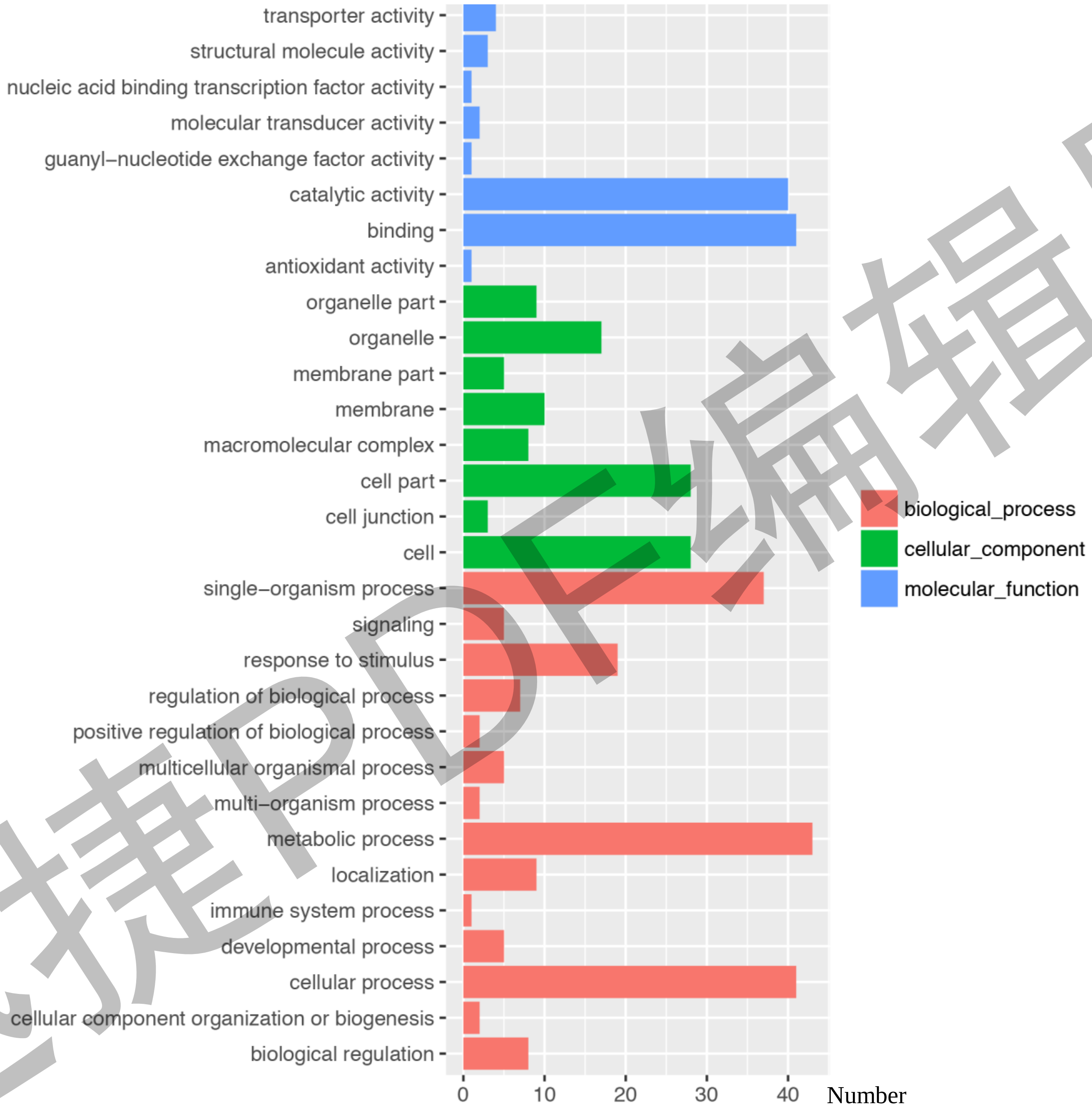

Supplementary Figure 9 Pathway analysis of *Verticillium dahliae* related genes identified

迅捷PDF编辑器

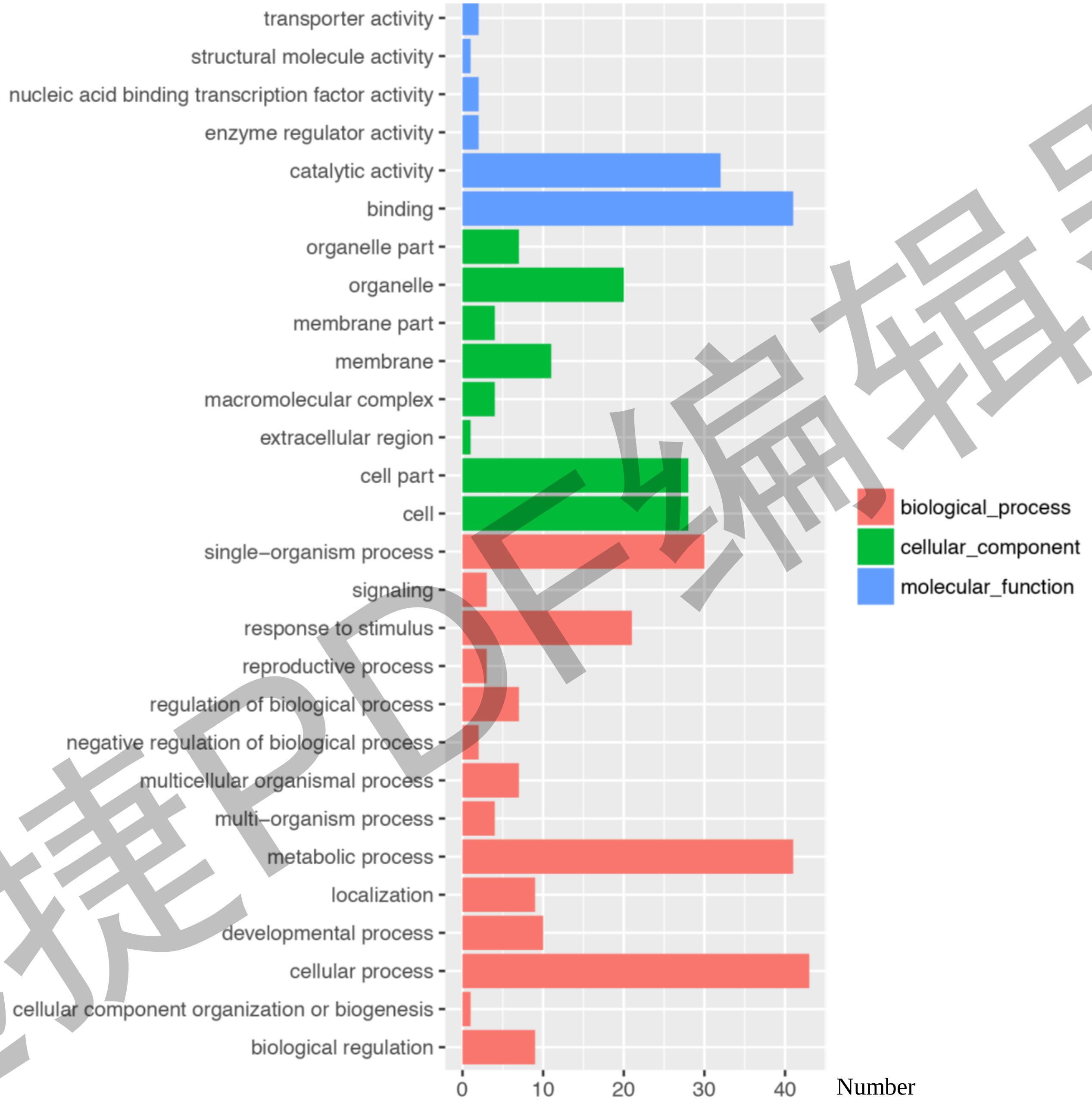

迅捷PDF编辑器

Supplementary Figure 10 Pathway analysis of salt related genes identified

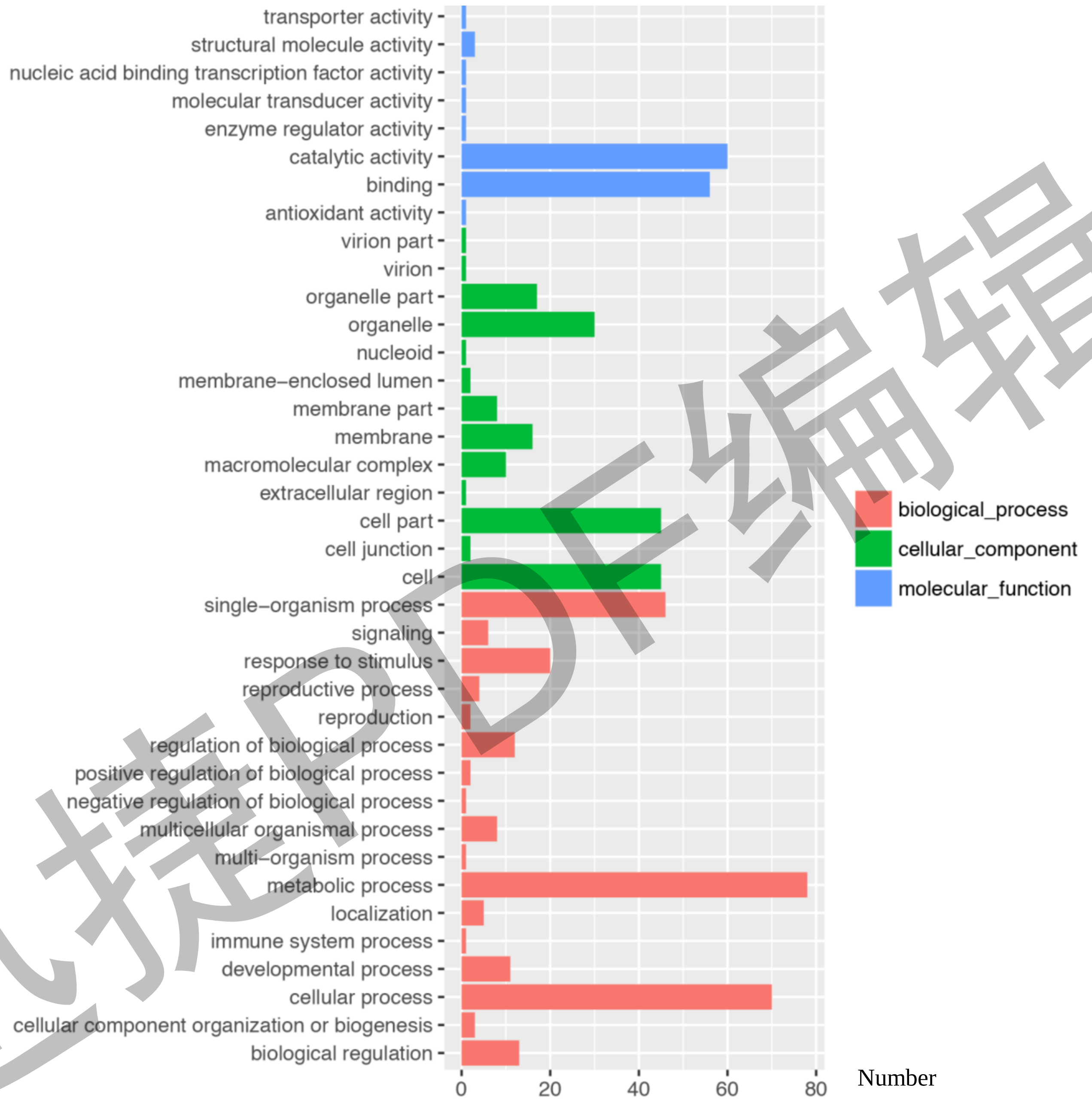

Supplementary Figure 11 Pathway analysis of drought related genes identified

迅捷PDF编辑器
